# Modelling the impact of insecticide-based control interventions on the evolution of insecticide resistance and disease transmission

**DOI:** 10.1186/s13071-018-3025-z

**Published:** 2018-08-28

**Authors:** Susana Barbosa, Katherine Kay, Nakul Chitnis, Ian M. Hastings

**Affiliations:** 1 Parasitology Group, Liverpool School of Tropical Medicine, L3 5QA, Liverpool, UK; 2 Present address: Université Côte d’Azur, Centre National de la Recherche Scientifique, Institut de Pharmacologie Moléculaire et Cellulaire, Valbonne, France; 3 Present address: Department of Pharmaceutical Sciences, State University of New York at Buffalo, Buffalo, New York 14214 USA; 4 Department of Epidemiology and Public Health, Swiss Tropical and Public Health Institute, Socinstrasse 57, 4002 Basel, Switzerland; 5 University of Basel, Petersplatz 1, 4003 Basel, Switzerland

**Keywords:** Insecticide, Resistance, Modelling, Malaria, Epidemiology, Mosquito

## Abstract

**Background:**

Current strategies to control mosquito-transmitted infections use insecticides targeted at various stages of the mosquito life-cycle. Control is increasingly compromised by the evolution of insecticide resistance but there is little quantitative understanding of its impact on control effectiveness. We developed a computational approach that incorporates the stage-structured mosquito life-cycle and allows tracking of insecticide resistant genotypes. This approach makes it possible to simultaneously investigate: (i) the population dynamics of mosquitoes throughout their whole life-cycle; (ii) the impact of common vector control interventions on disease transmission; (iii) how these interventions drive the spread of insecticide resistance; and (iv) the impact of resistance once it has arisen and, in particular, whether it is sufficient for malaria transmission to resume. The model consists of a system of difference equations that tracks the immature (eggs, larvae and pupae) and adult stages, for males and females separately, and incorporates density-dependent regulation of mosquito larvae in breeding sites.

**Results:**

We determined a threshold level of mosquitoes below which transmission of malaria is interrupted. It is based on a classic Ross-Macdonald derivation of the malaria basic reproductive number (R_0_) and may be used to assess the effectiveness of different control strategies in terms of whether they are likely to interrupt disease transmission. We simulated different scenarios of insecticide deployment by changing key parameters in the model to explore the comparative impact of insecticide treated nets, indoor residual spraying and larvicides.

**Conclusions:**

Our simulated results suggest that relatively low degrees of resistance (in terms of reduced mortality following insecticide contact) can induce failure of interventions, and the rate of spread of resistance is faster when insecticides target the larval stages. The optimal disease control strategy depends on vector species demography and local environmental conditions but, in our illustrative parametrisation, targeting larval stages achieved the greatest reduction of the adult population, followed by targeting of non-host-seeking females, as provided by indoor residual spraying. Our approach is designed to be flexible and easily generalizable to many scenarios using different calibrations and to diseases other than malaria.

**Electronic supplementary material:**

The online version of this article (10.1186/s13071-018-3025-z) contains supplementary material, which is available to authorized users.

## Background

Approximately 17% of human infectious diseases are transmitted by vectors such as mosquitoes, ticks and fleas [1, 2] and many are controlled by public health interventions using insecticides to target the vector. Malaria is the most serious example of a vector-borne infection and caused an estimated 212 million clinical cases and 429,000 deaths in 2016 [3]. Deploying insecticides against Anopheline mosquitoes, primarily in the form of insecticide-treated nets (ITNs) and indoor residual spraying (IRS), has been highly successful (see for example [4–7]) and are credited with contributing 68% and 13%, respectively, to recent dramatic reductions in falciparum malaria in Africa [7]. These successes come at a cost: large amounts of insecticides have to be deployed, and it is estimated that more than 50% of the population in sub-Saharan Africa was protected by at least one vector control intervention in 2015 [8]. A near-inevitable consequence has been the emergence and spread of insecticide resistance (IR) in mosquito vector species [9]. Almost two thirds of countries with ongoing malaria transmission now report resistance to one or more classes of insecticide [10–12] and this is widely recognised as a major threat to the sustainable impact of malaria control programmes (reviewed in [9]). Similar patterns of insecticide resistance are noted in other mosquito populations under public health control, notably the *Aedes* mosquitoes that transmit dengue.

The threat posed by insecticide resistance in mosquito populations has stimulated a series of theoretical papers to investigate the processes. They have been of two main forms. The first relates to evolutionary genetic and/or mathematical models exploring resistance management strategies designed to minimise selection for resistance (e.g. [13–20]). These models simply regarded insecticide resistance as something to be avoided and sought ways to understand, avoid or slow its evolution; this meant they usually had to ignore the most important operational factor of IR, i.e. its quantitative impact on undermining insecticide-based control of human disease transmission. A second suite of models does investigate the impact of insecticide resistance on mosquito population demography and hence on disease transmission (e.g. [19, 21, 22]). These could assess the impact of IR on control (using a ‘with’ *vs* ‘without’ comparison) but neglected the dynamics by which IR evolved and spread, and how it might be potentially delayed. The purpose of this paper is to close this methodological disconnect between the two approaches and demonstrate how they can be combined to simultaneously quantify the likely impact of insecticide deployment and resistance on malaria transmission potential.

We developed a demographic/genetic model for mosquito population dynamics that tracks overlapping generations and runs in discrete time steps of one day. It focuses on malaria transmission by its key vectors, *Anopheles*, although it can easily be modified to accommodate the bionomics of other species. The model incorporates the stage-structured mosquito life-cycle, i.e. eggs, larvae, pupae and adults. Modelling the adult stage allows mortality rates to differ between sexes (males do not blood-feed) and between the feeding and digesting/oviposition stages of the adult female. Density-dependent competition, and hence population regulation, is assumed to occur at the larval stage such that the emergence rate of new mosquitoes includes the non-linear impact of insecticides on reducing the population size. We integrated insecticide resistance into the model and allowed differential survival of mosquitoes depending on their genotypes (SS, SR and RR where S is the sensitive allele and R is the resistant), sex and the stage of the life-cycle (egg, larvae, pupae, adults). We then show how to interrogate this demography to calculate the R_0_ of the mosquito population; if vector R_0_ is less than 1 then the mosquito population will go extinct and disease transmission will cease. If extinction does occur we can then predict whether the presence (or importation) of resistance will be sufficient to re-establish the vector population, i.e. whether its R_0_ in the presence of resistance is greater than 1. We then used a Ross-Macdonald model to investigate situations where vector R_0_ > 1 to predict whether malaria transmission will continue despite control interventions reducing adult female population size and longevity and/or whether transmission will re-emerge once resistance is present in vector populations. The model is, therefore, designed to *simultaneously* answer a series of questions that arise naturally from control programmes:

- What impact do insecticides have on the mosquito population: will it be driven to extinction and, if not, how will insecticide deployment affect mosquito numbers and adult female longevity?
- What impact will these changes in mosquito demography have on disease transmission: assuming the mosquito populations are not eliminated, will there still be ongoing transmission?
- How will different patterns of insecticide deployment select for resistance?
- How will the spread of insecticide resistance affect mosquito populations and compromise attempts to reduce disease transmission?

We focus on malaria transmission, but Ross-Macdonald is a generic model for vector-borne disease transmission and, in principle, our methodology is equally applicable to other mosquito-borne diseases such as dengue.

## Methods

**Fig. 1:**
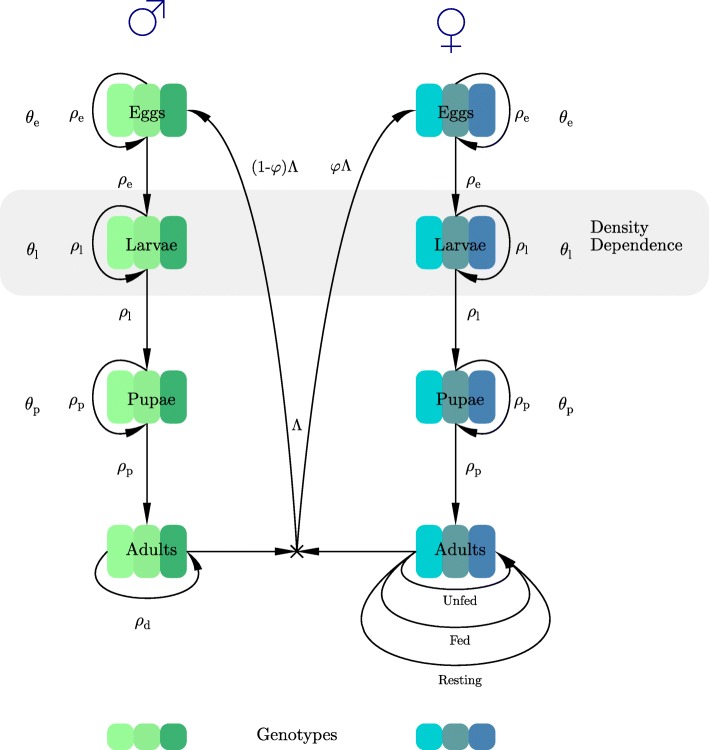
A schematic of our mosquito stage-structured model. The adult stage dynamics is considerably different in male and female mosquitoes primarily because male mosquitoes do not feed on vertebrate hosts and hence do not enter a host-seeking phase. Male adults are composed of newly emerged individuals plus the adult males that survived the previous day. Female adults are grouped in three classes: (i) unfed individuals that are currently host-seeking (newly emerged individuals, individuals that did not find a host the previous day, and individuals that laid eggs the previous day and are starting a new gonotrophic cycle), Eq. 12; (ii) fed individuals, Eq. 13; and (iii) resting individuals, Eq. 14. The model tracks the three potential genotypes *j* ∈ (*SS*, *RS*, *RR*) of the individuals through their developmental stages. The total number of eggs laid by all females is Λ (Eqs. 15 to 17), of which (1 − *φ*)Λ are males and *φ*Λ are females. We assume adult females mate once upon emergence, while males can mate multiple times. The *θ* parameters refer to the duration of each stage in days, and *ρ* to the proportion of individuals that survive per day in a given stage (*e*, eggs; *l*, larvae; *p*, pupae)

The anopheline mosquitoes that transmit malaria undergo complete metamorphosis through four distinct life-cycle stages: egg, larva, pupa and adult. Adult females feed on a vertebrate host and lay eggs in water bodies. Eggs hatch, within one or two days to a week or more, into larvae that breathe air through tubes, eating floating organic matter. Larvae moult four times until they became pupae. Pupae live near the surface of the water and do not eat, breathing through siphons on their back, and after a few days emerge as adults. The adult lives for a few days to several weeks [23]. The juvenile stages are similar in males and females, but the adult stage differs significantly in their behaviours as only females seek and feed on vertebrate hosts. A more detailed description of the life-cycle from a modelling perspective can be found in [24]. Note that because males do not bite and transmit infections they can be ignored in models that deal solely with transmission (e.g. [24, 25]) but they must be included here because they contribute half the genes to the next generation and their behaviour means adult males often inhabit a largely insecticide-free “refugia” with corresponding low selection for resistance [19]. Figure 1 outlines the model structure designed to reflect this life-cycle, and its parameterisation. It was constructed as a discrete-time, stage-structured model using a system of difference equations. The inclusion of the stage-structure allows realistic modelling of the life-cycle and selection of resistance at appropriate points within that life-cycle. Population regulation was assumed to occur through larval competition. The model was implemented in R [26] and used discrete time steps of one day to capture the circadian nature of mosquito behaviour.

We assumed resistance is encoded at a single gene with two alleles encoding resistance and sensitivity. We simultaneously ran this model in parallel for the three genotypes i.e. SS, SR and RR*.* This allows the genotypes to have different patterns of mortality depending on their level of insecticide resistance. Note that larval competition directly occurs between all three genotypes and that adults mate (at random) between the three genotypes. We assumed that males can mate multiple times but female mosquitoes mate once, immediately after emergence from pupae, and carry the sperm for the rest of their lives. We explicitly tracked the genotype of the sperm each female carries.

### A demographic/genetic model of mosquitoes under insecticide control

We use two superscripts in the notations: the first to denote gender (*f* for females and *m* for males) and the second to denote the mosquito genotype *j*, where *j* is one of SS, RS, or RR. We append a third superscript, *k*, to adult female mosquitoes, where *k* is one of SS, SR or RR and denotes the genotype of the male mosquito that she mated with. We describe the model parameters, and their specific values, for the life-cycle in Table 1.

**Table 1:** Parameters used in the mosquito demographic simulations

Symbol	Meaning	Default value (range)
*θ*_*e*_	Duration of the egg stage (days)	2
${\rho}_e^{fj}$ (${\rho}_e^{mj}\Big)$	Proportion of female (male) eggs of genotype *j* that survive one day	0.72 (0–1)
*θ*_*l*_	Duration of the larval stage (days)	10
${\rho}_l^{fj}$ (${\rho}_l^{mj}$)	Density-independent proportion of female (male) larvae of genotype *j* that survive one day	0.94 (0–1)
*Z*	Total larval resources availability (arbitrary units)	0.25 × 10^8^ (1 × 10^6^ – 0.33 × 10^11^)
${c}_i^{fj}$ (${c}_i^{mj}$)	Effect of larval competition on female (male) larvae of genotype *j* in stage *i*	0.67 (0–1)
${\omega}_i^{fj}$ (${\omega}_i^{mj}$)	Relative resource consumption of female (male) larvae of genotype *j* in stage *i*	0.67 (0–1)
*θ*_*p*_	Duration of the pupal stage (days)	3
${\rho}_p^{fj}$ $\left({\rho}_p^{mj}\right)$	Proportion of female (male) pupae of genotype *j* that survive one day	0.55 (0–1)
*τ*	Duration of the resting period of the gonotrophic cycle of a female adult mosquito (days)	3
*H* ^*j*^	Proportion of adult females of genotype *j* that find a host and successfully feed while seeking per day	0.67 (0–1)
${\rho}_s^{fj}$	Proportion of adult females of genotype *j* that survive while host-seeking per day	0.71 (0–1)
${\rho}_n^{fj}$	Proportion of adult females of genotype *j* that survive while resting per day	0.96 (0–1)
${\rho}_d^{mj}$	Proportion of male adults of genotype *j* that survive one day	0.5 (0–1)
*β* ^*j*^	Number of eggs laid per oviposit by female mosquitoes of genotype *j*	100
*σ* ^*k*^	Mating viability of a male of genotype *k*. 0 < *σ*^*k*^ ≤ 1	1.0
*φ*	Proportion of eggs that are female	0.5

The superscripts, *m* and *f* denote males and females; *j* and *k* denote genotype and can be any of *SS*, representing homozygous susceptible, *RS*, representing heterozygous, and *RR*, representing homozygous resistant. Default parameter values are identical for males and females when applicable. The range gives, where applicable, the values used in the sensitivity analysis and define the limits of a triangular (with mode 0.85) for all parameters except c, ω and *Z* that follow a uniform distribution. Parameter choice and supporting citations are provided and discussed in Additional file 3

#### Tracking the mosquito juvenile population

Development through the juvenile life-cycle is tracked using the index *i* to represent days since the egg was laid (*i* = 1 denotes a newly laid egg): *θ*_*e*_ is the duration of the egg stage, *θ*_*l*_ is the duration of the larval stage and *θ*_*p*_ is the duration of the pupal stage (all measured in days). The total duration of the juvenile stages is therefore *ζ* where *ζ* = *θ*_*e*_ + *θ*_*l*_ + *θ*_*p*_ and we denote the female juvenile mosquito population of genotype *j* at time *t* as *x*^*fj*^(*t*) where

- ${x}_i^{fj}(t)$ for 1 ≤ *i* ≤ *θ*_*e*_ denotes the number of female egg stages, of genotype *j*, of age *i*, at time *t*,
- ${x}_i^{fj}(t)$ for (*θ*_*e*_ + 1) ≤ *i* ≤ (*θ*_*e*_ + *θ*_*l*_) denotes the number of female larval stages of genotype *j*, of age *i*, at time *t*,
- ${x}_i^{fj}(t)$ for (*θ*_*e*_ + *θ*_*l*_ + 1) ≤ *i* ≤ *ζ* denotes the number of female pupal stages of genotype *j*, of age *i*, at time *t*.

The male juvenile population is described in an analogous manner with a superscript *m* instead of *f*. Note that the symbol ${x}_i^{--}(t)$ denotes the number of juveniles at stage “i” at the end of day “t”. The equations in this section therefore all function in the same way. They calculate the number of mosquitoes coming into stage “i” at the start of the current day [i.e. from the previous day and stage, ${x}_{i-1}^{--}\left(t-1\right)$], allowing for factors such as density dependence and mating, and then multiplying this number by the survival probability of that stage to obtain the number surviving at the end of that day, i.e. ${x}_i^{--}(t)$.

We describe the dynamics of the juvenile male and female mosquito populations of genotype *j* in Eqs. 1 to 8. After each iteration, mosquitoes are moved forward in chronological time (to *t* + 1) and in developmental time (to *i* + 1).

The juvenile female mosquito population of genotype *j* at time *t*, *x*^*fj*^(*t*) was tracked by first determining the number of newly laid female eggs i.e. the first day of the egg stage, *i* = 1:

1 $$ {x}_1^{fj}(t)={\Lambda}^j\left(t-1\right)\varphi {\rho}_e^{fj} $$

where Λ^*j*^(*t* − 1) is the total number of eggs of genotype *j* laid at time *t* – 1 (see later discussion of Eqs. 15 to 17) and *φ* is the proportion of female eggs (always set to 0.5 here).

The developing eggs after the first day were tracked using:

2 $$ {x}_i^{fj}(t)={x}_{i-1}^{fj}\left(t-1\right){\rho}_e^{fj}\mathrm{for}\ 2\le i\le {\theta}_e $$

where eggs develop over θ_*e*_ days and progress is dependent on the daily egg survival probability, *ρ*_*e*_.

The larval stages were tracked as:

3 $$ {x}_i^{fj}(t)={x}_{i-1}^{fj}\left(t-1\right)\left[\frac{1}{1+{c}_i^{fj}\ \frac{L\left(t-1\right)}{Z}}\right]{\rho}_l^{fj}\mathrm{for}\ \left({\theta}_e+1\right)\le i\le \left({\theta}_e+{\theta}_l\right) $$

where larval stages persist for *θ*_*l*_ days and progress is dependent on the daily larval survival probability *ρ*_*l*_. In this model, density-dependent population regulation (DDPR) occurs in the larval stages of both sexes and is represented by the factor encoded in square brackets. This factor is described in more detail below in Eqs. 9 and 10.

The pupal stages were tracked as:

4 $$ {x}_i^{fj}(t)={x}_{i-1}^{fj}\left(t-1\right){\rho}_p^{fj}\mathrm{for}\ \left({\theta}_e+{\theta}_l+1\right)\le i\le \zeta $$

The juvenile male mosquito population of genotype *j* at time *t*, *x*^*mj*^(*t*) was similarly defined for number of male eggs, developing eggs, larval stages and pupal stages as

5 $$ {x}_1^{mj}(t)={\Lambda}^j\left(t-1\right)\left(1-\varphi \right){\rho}_e^{mj} $$

6 $$ {x}_i^{mj}(t)={x}_{i-1}^{mj}\left(t-1\right){\rho}_e^{mj}\mathrm{for}\ 2\le i\le {\theta}_e $$

7 $$ {x}_i^{mj}(t)={x}_{i-1}^{mj}\left(t-1\right)\left[\frac{1}{1+{c}_i^{mj}\ \frac{L\left(t-1\right)}{Z}}\right]{\rho}_l^{mj}\mathrm{for}\ \left({\theta}_e+1\right)\le i\le \left({\theta}_e+{\theta}_l\right) $$

8 $$ {x}_i^{mj}(t)={x}_{i-1}^{mj}\left(t-1\right){\rho}_p^{mj}\mathrm{for}\ \left({\theta}_e+{\theta}_l+1\right)\le i\le \zeta $$

#### Implementing density-dependent population regulation (DDPR)

The DDPR was incorporated into the larval populations in Eqs. 3 and 7 using the Leslie Gower population growth model, analogous to Beverton-Holt (B-H), which is a classic discrete time population growth model whose continuous-time equivalent is logistic growth towards a carrying capacity [27]. The B-H equation is:

9 $$ {x}_{t+1}={x}_t{R}_0\left[\frac{1}{1+\frac{x_t}{Z}}\right] $$

where *x*_*t*_ is the number of individuals at generation *t*, *R*_0_ is the per capita growth rate per generation and *Z* is a number that determines the carrying capacity of the population, *K*, as *K* = (*R*_0_ − 1)*Z*. We extend the B-H model in Eqs. 3 and 7 with a change in scale from individual animals (*Z* in Eq. 9) to amount of larval resources to account for competition between different genotypes (in this manuscript). The DDPR described within square brackets in Eqs. 3 and 7 is analogous to that in Eq. 9 with this change of scale. The total amount of larval resources is user-defined as a constant *Z* in arbitrary, undefined units, which sets the carrying capacity of the population. *L*(*t*) is the current amount of larval resources being consumed at time *t* (see below) hence the ratio *L*(*t*)/*Z* in Eqs. 3 and 7 plays exactly the same role as *x*(*t*)/*Z* in Eq. 9; it is simply that Eq. 7 defines the approach to carrying capacity in units of resources while Eq. 9 defines it in units of population. The only remaining difference between Eqs. 7 and 9 is the extra term *c* in Eq. 7 that describes relative competitive ability of the genotypes, age and sex of the larvae. The competitive ability of larvae, ${c}_i^{fj}$ and ${c}_i^{mj}$, may differ depending on their genotype (for example, resistant forms may pay a fitness penalty for carrying the resistance mutation) and the resource consumption (denoted *ω*^*fj*^ and *ω*^*mj*^, see below) of each genotype may vary (for example, resistant forms may be larger and consume more resources). Similarly, older larvae are likely to consume more food and may be more resilient to competition. The total larval consumption of resources by male and female larvae of all genotypes is obtained simply by summing over the sexes, genotypes, and stages, i.e.

10 $$ L(t)=\sum \limits_{j\in \left\{ SS, RS, RR\right\}}\left(\sum \limits_{i={\theta}_e+1}^{\theta_e+{\theta}_l}{\omega}_i^{fj}{x}_i^{fj}(t)+{\omega}_i^{mj}{x}_i^{mj}(t)\right) $$

where *ω*_*i*_ is the relative resource consumption of the larval sex/genotype, the latter being indicated by its superscript, *j*, of age *i*. Isolating the DDPR as a distinct factor in Eqs. 3 and 7 means it is simple to substitute other forms of DDPR if required (e.g. [24, 25]) or other functions such as the Ricker function [28].

#### Tracking the mosquito adult population

The adult male population of genotype *j* at time *t*, *y*^*mj*^(*t*) is:

11 $$ {y}^{mj}(t)=\left[{y}^{mj}\left(t-1\right)+{x}_{\zeta}^{mj}\left(t-1\right)\right]{\rho}_d^{mj} $$

which is the number of male adults that survived from the previous day (*y*^*mj*^(*t* − 1)) augmented by male adults that emerged from pupae $\left({x}_{\zeta}^{mj}\left(t-1\right)\right)$, scaled by the probability that they survive the day (${\rho}_d^{mj}$).

Females mate once when they emerge and store the sperm to fertilise all their future egg production while males may mate multiple times (see for example [29]). Female anophelines need to blood-feed to produce eggs, so their behaviour differs significantly from those of males (who do not blood-feed). Fertilised females initiate their gonotrophic cycle that consists of 3 phases: (i) foraging for a host and blood-feeding; (ii) resting to allow digestion of the blood and egg maturation; and (iii) searching for a suitable oviposition site and oviposition (Fig. 1). This gonotrophic cycle is repeated throughout the female’s remaining lifespan.

The female adult population time *t* + 1 is described in Eqs. 12 to 14. Recall that adult female mosquitoes require a third superscript *k* (where *k* is one of SS, SR or RR) to denote the genotype of the male mosquito she mated with (which will be the paternal genotype for her subsequent egg production).

The number of host-seeking unfed females in the current gonotrophic cycle is:

12 $$ {y}_1^{fj k}(t)=\kern0.5em \left[{x}_{\zeta}^{fj}\left(t-1\right)\frac{\sigma^k\left({y}^{mk}\left(t-1\right)+{x}_{\zeta}^{mk}\left(t-1\right)\right){\rho}_d^{mk}}{\sum_{h\varepsilon \left\{ SS, RS, RR\right\}}{\sigma}^H\left({y}^{mk}\left(t-1\right)+{x}_{\zeta}^{mk}\left(t-1\right)\right){\rho}_d^{mk}}+\kern0.5em {y}_1^{fj k}\left(t-1\right)\left(1-{H}^j\right)\kern0.5em +\kern0.5em {y}_{\tau}^{fj k}\left(t-1\right)\right]{\rho}_s^{fi} $$

where the first term describes the number of newly emerged female adults *f*, of genotype *j* $\left({x}_{\zeta}^{fj}\left(t-1\right)\right)$, that will mate with a male of genotype *k* $i.e.\left(\ {y}^{mk}\left(t-1\right)+{x}_{\zeta}^{mk}\left(t-1\right)\right){\rho}_d^{mk}$, which has a mating viability *σ*^*k*^ (this term is normalised by dividing by the total adult male population weighted by their mating viability). The second term, ${y}_1^{fjk}\left(t-1\right)\left(1-{H}^j\right)$, refers to other female mosquitoes still in the host-seeking state that were unfed adults the previous day and unsuccessful in finding a host on the previous day (*H* is the probability of successfully finding a host and feeding). The third term, ${y}_{\tau}^{fjk}\left(t-1\right)$ represents females that successfully laid eggs, completing their gonotrophic cycle, and are now seeking a host in their new gonotrophic cycle. These terms are then scaled by the probability that they survive this day of host-seeking, i.e.${p}_s^{fj}.$

The number of female mosquitoes entering the second adult phase of the gonotrophic cycle (resting and fed the previous day) corresponds to individuals in *y*_1_ that survived and successfully fed (a proportion *H*^*j*^) and is described as:

13 $$ {y}_2^{fj k}(t)={y}_1^{fj k}\left(t-1\right){H}^j{p}_n^{fj} $$

The number of females in the remaining days of this “resting” phase of digestion of the blood and egg maturation, was found using:

14 $$ {y}_i^{fj k}(t)={y}_{i-1}^{fj k}\left(t-1\right){\rho}_n^{fj}\mathrm{for}\ 3\le i\le \tau $$

if the duration of the resting stage is sufficiently long i.e. (*τ* ≥ 3).

We assume, for simplicity, that the probability rested females successfully survive while finding an oviposition site and mating (the third phase of the female gonotrophic cycle) is the same as their daily probability of survival while resting, i.e. ${\rho}_n^j$. This factor enters the equations describing egg laying, i.e. Eqs. 15 to 17 below.

#### Tracking the spread of resistance

The frequency of resistance is defined at the start of the simulations and is assumed to be equal in males and females, with genotypes in Hardy-Weinberg equilibrium [30]. Male and female genotypes are tracked separately in the simulations (Fig. 1) because their exposure to insecticides as adults will differ and hence genotype frequencies may differ between males and females in the adult, breeding population. Mating is assumed to occur at random and inheritance is by standard Mendelian genetics. We can therefore calculate the proportion of genotypes in the next generation according to the following three equations where *β*^*j*^ is the number of eggs laid by genotype *j*.

The number of homozygous susceptible eggs laid at time *t* is

15 $$ {\Lambda}^{SS}(t)={\beta}^{SS}{\rho}_n^{SS}\left({y}_{\tau}^{fSSSS}(t)+\frac{1}{2}{y}_{\tau}^{fSSRS}(t)\right)+{\beta}^{RS}{\rho}_n^{RS}\left(\frac{1}{2}{y}_{\tau}^{fRSSS}(t)+\frac{1}{4}{y}_{\tau}^{fRSRS}(t)\right) $$

The number of heterozygous eggs laid at time *t* is

16 $$ {\wedge}^{RS}(t)=\kern0.5em {\beta}^{SS}{\rho}_n^{SS}\left(\frac{1}{2}{y}_{\tau}^{fSSRS}(t)+\kern0.5em {y}_{\tau}^{fSSRR}(t)\right)+{\beta}^{RS}{\rho}_n^{RS}\left(\frac{1}{2}{y}_{\tau}^{fRSSS}(t)+\frac{1}{2}{y}_{\tau}^{fRSRS}(t)+\frac{1}{2}{y}_{\tau}^{fRSRR}(t)\right)+{\beta}^{RR}{\rho}_n^{RR}\left({y}_{\tau}^{fRRSS}(t)+\frac{1}{2}{y}_{\tau}^{fRRRS}(t)\right) $$

The number of homozygous resistant eggs laid at time *t* is

17 $$ {\Lambda}^{RR}(t)={\beta}^{RS}{\rho}_n^{RS}\left(\frac{1}{4}{y}_{\tau}^{fRSRS}(t)+\frac{1}{2}{y}_{\tau}^{fRSRR}(t)\right)+{\beta}^{RR}{\rho}_n^{RR}\left(\frac{1}{2}{y}_{\tau}^{fRRRS}(t)+{y}_{\tau}^{fRRRR}(t)\right) $$

The *ρ* parameters in these equations represent the additional mortality associated with searching for oviposition sites; for simplicity, these were assumed to be equal to that of non-host-seeking.

Immigration, emigration and mutation are absent but it would be straightforward to include these effects by altering genotype frequencies at the egg stage (mutations) or by altering the number and/or genotypes of adult stages to represent immigration/emigration.

### Estimating population basic reproductive rate (R_0_) for mosquitoes

A natural question considered by control programmes is whether an intervention will eliminate the local mosquito population. It is possible to run the model described above to find if a population is viable, i.e. start the demographic simulation from extremely low mosquito numbers and find if they increase over the longer term and a stable age distribution has been reached. This is computationally expensive, especially if large-scale sensitivity analyses are being run, so an algebraic expression for R_0_ is desirable. The *R*_0_ for female mosquitoes, ignoring differences in genotypes, is

18 $$ {R}_0=\frac{\varphi \beta {\rho}_e^{\theta_e}{\rho}_l^{\theta_l}{\rho}_p^{\theta_p}{\rho}_sH{\rho}_n^{\tau -1}}{1-{\rho}_s\left(1-H\right)-{\rho}_sH{\rho}_n^{\tau -1}} $$

This equation can be derived in two ways (using an “intuitive” approach and a rigorous mathematical approach); both yield the same result and are described in Additional files 1 and 2, respectively. Obviously if R_0_ < 1, the mosquitoes are locally extinct and no disease transmission will occur, i.e. the intervention has succeeded.

### Estimating population basic reproductive rate (R_0_) for malaria and human malaria prevalence

Assuming a viable mosquito population remains despite the intervention (i.e. R_0_ > 1 for mosquitoes, see above), the next step is to predict whether this mosquito population is able to transmit malaria. The basic reproductive rate of malaria, *R*_*0m*_, using the approach attributed to Ross and Macdonald (R-M) [31] is as follows (although we note there are several variations of this basic equation [32]):

19 $$ {R}_{0m}=\frac{m{a}^2{b}_1{b}_2}{gr}{\rho}_i=\frac{M}{N}\bullet \frac{a^2{b}_1{b}_2}{gr}{\rho}_i $$

where:

- *m=M/N* is the number of female mosquitoes per human host where *N* is the size of the human population and *M* is the size of the female adult mosquito population (i.e. *A*_*f*_(*t*) in our models, see later description of Eq. 23);
- *a* is the rate of biting on humans by a single mosquito (number of bites per unit time);
- *b*_1_ is the probability of infection transmission from infectious mosquitoes to susceptible humans;
- *b*_2_ is the probability of infection transmission from infectious humans to susceptible mosquitoes
- *r* is the per capita rate of recovery for humans (so 1/*r* is the average duration of infection in the human host);
- *g* is the per capita constant mortality rate for female mosquitoes (so 1/*g* is the average life time of a mosquito). This is usually obtained as − ln(*p*) where *p* is the daily survival rate (Box 2 of [32]);
- *ρ*_*i*_ is the probability of surviving the “extrinsic incubation period” i.e. the time period between the mosquito biting a malaria-infected human and that mosquitoe becoming infectious to other humans (i.e. the presence of sporozoites in her mouthparts).

The R-M approach does not differentiate between females in different stages of their gonotrophic cycle whereas our approach explicitly defines different death rates according to the behaviour of the female of any given day (i.e. actively host-seeking or resting). We now illustrate how the R-M approach may be used with differential female survivorship in different states. We will assume, for convenience, locally intense transmission of malaria by *An. gambiae*, a vector that feeds almost exclusively on humans and bites approximately every 4 days (see Additional file 3). Assuming the female always completes her cycle in these 4 days and then proceeds to the next cycle, the approximate adult female daily survival probability is the geometric mean of daily survival rates during the cycle, so that the daily mortality rate is:

20 $$ g=-\mathit{\ln}\left[\sqrt[4]{\rho_s^j{\left({\rho}_n^j\right)}^3}\right]=-\mathit{\ln}\left[{\rho}_s^j{\left({\rho}_n^j\right)}^3\right]/4 $$

The duration of the extrinsic incubation period depends on temperature but assuming ideal conditions, we will let it be ~10 days; we also assume that the adult always finds a human on the day she starts host-seeking. The mosquitoes will have fed at the start of the extrinsic incubation period so the extrinsic incubation period will consist of 3 days resting, another day host-seeking, 3 days resting, another day feeding and 3 days resting until she is ready to feed again and transmit the infection, i.e. 9 days resting and 2-host-seeking so we can estimate the probability of surviving the extrinsic incubation period as:

21 $$ {\rho}_i={\left({\rho}_n^j\right)}^9{\left({\rho}_s^j\right)}^2 $$

These calculations were, as mentioned above, based on ideal situations for mosquitoes [i.e. they always find hosts (so *H* = 1), extrinsic incubation only lasts 10 days, and so on]. Mosquitoes can be age-dated by parity in the field so we can revise Eq. 21 to obtain the probability a mosquito survives 3 or more feeding/parity cycles, each cycle of 3 days resting and one feeding, as (0.96^3^ × 0.71)^3^ = 0.25. This is rather high but not unrealistic. Gilles & Wiles [33] found 20% and 23% of *An. gambiae* and *An. funestus*, respectively, were “3-parous *and older*” in Muheza, Tanzania; these data came from 1965 when there was much less insecticide being deployed in public health. Background mortality rates will be higher in contemporary settings with widespread insecticide deployment although incorporating this background exposure greatly complicates extraction of basal mortality rates (see [34] for a recent example). More realistic calculations may be used to incorporate ‘non-perfect’ conditions in the mosquito populations, for example wide scale ITN coverage may mean mosquitoes take two or even three days of host-searching to obtain a blood meal. Equations 20 and 21 can be updated to reflect these new combinations of days spent searching and resting. In reality, a range of different combinations will occur in the mosquito population and the solutions to the equations will be a type of weighted mean across the combinations [35]. We omitted these complications in the interests of simplicity, because the calculations only serve as illustrative target reductions for interventions (see later) and to avoid duplication of previous work [35].

This approach does enable us to obtain the parameters required to calculate, M’, the target number of adult mosquitoes that results in *R*_*0m*_ < 1 and hence elimination of malaria as

22 $$ {M}^{\prime }<N\frac{rg}{a^2{b}_1{b}_2{\rho}_i} $$

These equations do not distinguish between the genotypes with differing levels of insecticide resistance (which results in the different genotypes having different survival probabilities). It is straightforward to incorporate resistant genotypes by regarding them as equivalent to different “species”; since malaria is often spread by more than one vector species, methods for calculating *R*_0_ in the presence of several species is well worked out [36, 37].

The value of R_0m_ allows the equilibrium prevalence, $\widehat{P}$ of malaria in humans to be calculated algebraically. Anderson & May [31], for example, calculated it as $\widehat{P}=\left({R}_0-1\right)/\left({R}_0+a{b}_2/g\right)$. The problem with R-M applied to malaria is that it does not allow for super-infection or for acquired immunity so prevalences obtained algebraically should be interpreted with caution. An alternative, and probably more robust approach, is to obtain R_0m_ as described above and then obtain malaria prevalence using their empirical relationships estimated from field surveys; for example, using figure 2 of [38] to convert R_0_ to entomological inoculation rate (EIR) and then figure 1 of [39] to convert EIR to prevalence.

**Fig. 2:**
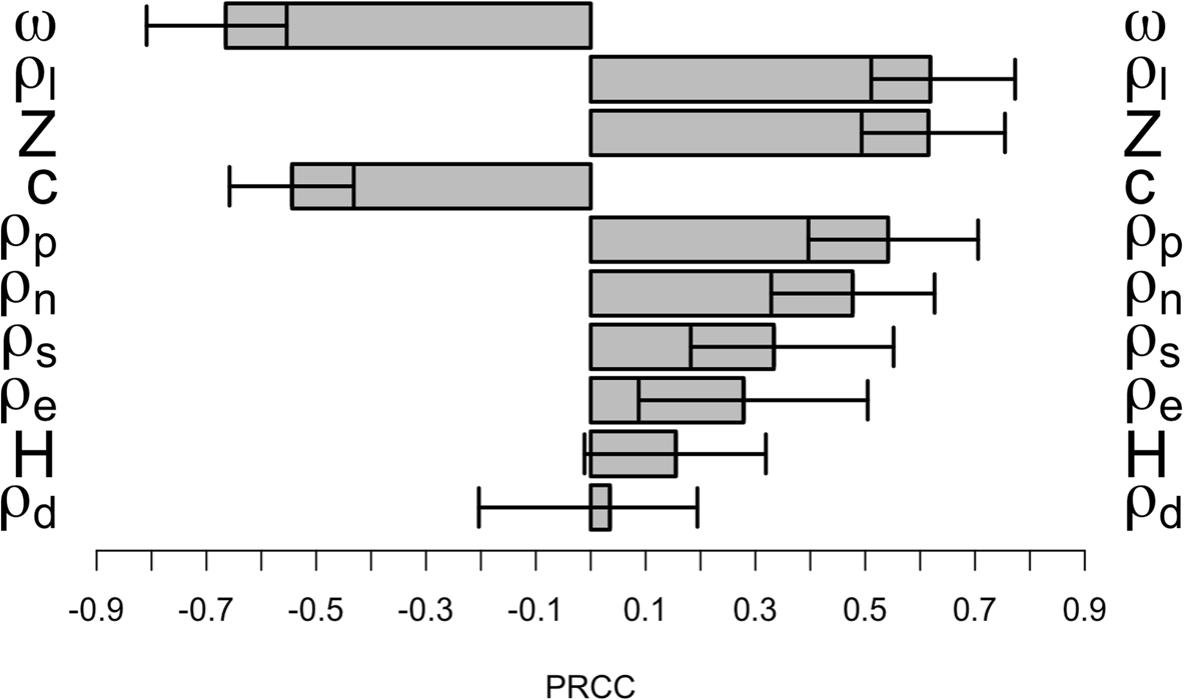
Partial rank correlation coefficients (PRCC) between equilibrium adult female population size and selected model parameters. The parameters symbols in the y-axes are defined in Table 1 and the horizontal error bars delimit the 95% confidence intervals. In order of importance: ω is resource consumption of larvae, *ρ*_*l*_is larval survival (per day), *Z* is total larval resources, *c* is the impact of larval competition, *ρ*_*p*_is pupal survival (per day), *ρ*_*n*_is adult female survival (per day) when resting, *ρ*_*s*_is adult female survival (per day) when host-seeking, *ρ*_*e*_is egg survival (per day), *H* is the proportion of adult females that successfully find a host (per day), and *ρ*_*d*_is adult male survival (per day)

### Simulating mosquito populations

When tracking the spread and impact of resistance in the simulation, the starting frequency of resistance was assumed to be 0.5 in all cases. The initial frequencies will be much lower at the start of most real-life interventions so these simulations show the last stages of resistance spread following interventions. These intermediate frequencies of alleles reduce the impact of stochastic frequency changes, allowing better estimation of selection coefficients; these coefficients are key summary measures in population genetic theory allowing results to be generalised. For example, section coefficients determine the rate of geographical migration of resistance, and the chance of resistance alleles first emerging in the populations (e.g. [40]). The genotypes were introduced in Hardy-Weinberg equilibrium, i.e. 25, 50 and 25% of the SS, SR and RR genotypes, respectively. We calibrate the models for an area of high malaria transmission and use a value of 110 for mosquito density (i.e. number of adult female mosquitoes per human host; *m* in the Ross-Macdonald model) as justified in Additional file 3.

A useful starting point for identifying high-impact interventions is to use the calculations developed above to identify those parameters, such as larval survival probability, in which small changes may have a disproportionately large impact on adult female population size. This enables us to identify key parameters which are prime candidates to be targeted by insecticides. The demographic/genetic model described above was run to equilibrium adult population size using 3000 randomly generated combinations of parameter values drawn from the parameter space described in Table 1 with no genetic differences in resistance levels. The output allowed us to perform a sensitivity analysis of the influence of the parameters on female population size. The total number of adult females at time *t*, *A*_*f*_(*t*) is the sum of the number of mosquitoes in each day of the feeding cycle as described by Eqs. 12 to 14, i.e.

23 $$ {A}_f(t)=\sum \limits_{i=1}^{\tau}\kern0.5em \sum \limits_{j\in \left\{ SS, SR, RR\right\}}\sum \limits_{k\in \left\{ SS, SR, RR\right\}}{y}_i^{fjk}(t) $$

Mann-Witney and t-tests were used to compare the mean parameter values that generated a viable malaria population with those that lead to extinction. Partial rank correlation coefficients (PRCC) were then calculated as a sensitivity analysis of the model using only the simulations that generated viable populations. The PRCC were only calculated between parameters expected to be affected by vector control measures (i.e. *ρ*_*e*_, *ρ*_*l*_, *ρ*_*p*_, *ρ*_*s*_, *ρ*_*n*_, *c*, *ω*, *Z*, *ρ*_*d*_ and *H*, assuming no differences between males or females in parameter values in the non-adult stages); the magnitude of the absolute PRCC values can be used to rank the relative importance of the 10 input parameters.

### The impact of insecticide deployment and threat posed by resistance

Our main goal with the development of this model was to address operational issues of insecticide deployment and how it both drives, and is compromised by, resistance. We focus on exploring the generic issues concerning the application of insecticides rather than attempting to parameterise a particular setting because there are limited data on many of the key parameters, particularly for differential survival of the different sensitive/resistant genotypes. The combination of default parameters given in Table 1 resulted in a viable population in the absence of insecticide deployment; we then investigated the likely impact of insecticide deployment (and resistance) by changing the values of parameters that are likely to be affected by the intervention. In particular, we ran simulations that mimic larvicides, ITNs and IRS and their impact on the mosquito population.

The total adult female mosquito population at equilibrium (Eq. 23) for the default parameters in Table 1 is *A*_*f*_ = 135,878. The equivalent number of humans for this default setting was then obtained as *N* = 1235 using the value of *m* = 110 (Additional file 3). It is now possible to use this value of *N* together with the numbers of adult female mosquitoes when under control measures to obtain *M*^′^ using Eq. 22 and hence to predict whether disease transmission is possible.

We then investigated how the emergence and spread of resistance would impact insecticide-based interventions and, in particular, whether the spread of resistance would allow mosquito populations to recover to the extent that malaria transmission would restart, i.e. *R*_0*m*_ > 1. We present the worst-case scenarios in terms of spread of resistance, because we assume resistance to be completely dominant, i.e. we assume the survival probabilities of the heterozygote and homozygote resistant genotypes to be equal.

When considering the interventions below we assume that those targeting the non-adult stages have the same impact on both males and females as there is little, if any, sexual difference in exposure in these stages, e.g. an intervention reducing the female larvae daily survival probability by 50% would also reduce male larval survival probability by 50%. Interventions targeting adults are assumed to only affect females. This avoids having to define a differential impact on the two sexes that will almost certainly arise due to behavioural differences; for example, IRS may reduce adult female resting survival by 80% but adult male survival by only 5%. Defining this differential impact on adults is also unnecessary because male adult survival has no impact on overall population size (because all females are assumed to mate successfully irrespective of male population size). Ignoring the potential impact of IRS and ITN on male mortality slows the rate at which resistance spreads (because there is no selective pressure on males by IRS or ITN) but we regard this as a reasonable simplification that could be relaxed later. Note that we do include male mortality at the larvae stages because they contribute to DDPR; their deaths lessen competition at this stage and help reduce the impact of female larval mortality on the eventual adult population size.

#### Single-insecticide interventions

We initially simulate the dynamics of the mosquito population under reduced survival imposed by the use of insecticides that target single stages of the life-history and without the emergence of resistance. We use the default values given on Table 1 as the baseline values that lead to a viable population. We assume that ITNs act by decreasing the survival probability of female adults while host-seeking, *ρ*_*s*_; IRS reduces female adult survival while resting, *ρ*_*n*_; larvicides reduce the survival probabilities of larvae, *ρ*_*l*_; and a pupacide that kills only pupae, *ρ*_*p*_ (we are unaware of any agents that do this but include this hypothetical example for methodological completeness). Henceforth we will be using the intervention name and the parameter that we assume it affects interchangeably.

We reduced each survival probability by 10, 30, 40 and 80% of the original value to explore the impact of different degrees of intervention effectiveness. We tracked the number of adult female mosquitoes post-intervention and the intervention was considered to be successful if the number of females was reduced below a threshold value obtained using Eq. 22, below which malaria transmission would be theoretically interrupted.

#### Combined-insecticide interventions

Interventions often use combinations of insecticides that target two or more stages of the mosquito life-history. The impact of these interventions was investigated, as for single interventions, by reducing the survival probabilities of the affected life-stages by 10%, 30%, 40% and 80% of the original value and tracking the number of adult female mosquitoes. We investigated three specific interventions as listed below. They are designed to illustrate our approach rather than to simulate specific, well-calibrated examples (see later discussion around calibration). The interventions are as follows:

- ITNs and IRS: these interventions reduce the survival probabilities of host-seeking adult females (ITN: *ρ*_*s*_) and resting females (IRS: *ρ*_*n*_).
- Larviciding and IRS: larviciding (for example with temephos) is assumed to affect both larvae and pupae (*ρ*_*l*_ and *ρ*_*p*_) while IRS, as above, reduces the survival of non-host-seeking adult females (*ρ*_*n*_)
- Larviciding and ITNs: as above, larviciding is assumed to reduce *ρ*_*l*_ and *ρ*_*p*_ while ITN reduces the survival of host-seeking adult females, *ρ*_*s*_.

The combination of interventions was considered successful if the number of females was reduced below the critical threshold value below which malaria transmission is theoretically interrupted.

## Results

We ran the model using 3000 randomly generated combinations of parameters from the distributions described in Table 1 which resulted in viable mosquito populations at equilibrium in 103 (3.4%) of these runs. Statistical analysis (two-tailed t-tests and Mann-Witney U-tests) on the parameters used in the sensitivity analysis (Table 1) showed that the following parameters were highly significantly (*P* < 0.0001 in both tests after correcting for multiple testing using the BH method) higher in simulations that resulted in viable populations compared to those that went extinct: the daily survival probabilities of the immature stages (i.e. eggs, larvae and pupae), of females seeking a host, and of females resting (*ρ*_*e*_, *ρ*_*l*_, *ρ*_*p*_, *ρ*_*s*_, *ρ*_*n*_ respectively). In contrast, parameters describing the effect of larval competition (*c*), relative resource consumption (*ω*), resource availability (*Z)*, the daily probability that a female successfully finds a host and feeds (*H*), and the proportion of male mosquitoes (*ρ*_*d*_) were not statistically different (*P* > 0.05). Among the non-significant parameters, the first three are associated with the larval competition that is absent when populations are at very low densities and are therefore expected to have no effect on determining whether a population is viable or not (although they will, of course affect the equilibrium size of viable populations). The fourth factor, *H*, is non-significant, presumably because a female that fails to find a host one day can survive and successfully feed the next. Finally, *ρ*_*d*_ is daily adult male survivorship which again is not expected to affect whether a population is viable because we assume females always find a male and that males can mate multiple times.

These t-tests and Mann-Witney tests reveal whether a factor has an impact on whether a mosquito population is viable, but it is the PRCC analyses that reveal the parameters with the largest impact in determining the size of the adult female population. Density-dependent population regulation in our models is assumed to occur by larval competition so it is not surprising that those factors with the largest impact on adult population size were those controlling the intensity of larval competition, i.e. the total larval resources available (*Z*), the relative resource consumptions of larvae (*ω*), the impact of larval competition (c), and daily larval survivorship (*ρ*_*l*_), see Fig. 2. Note that the PRCC results on final population size are consistent with the t-test and Mann-Whitney results described in the previous paragraph on whether a population is viable, i.e. the daily survival probabilities are all highly significant (with the except of male survival) while the probability that a female successfully finds a host and feeds (*H*) and male survival are non-significant. The only difference is in factors associated with resource availability (*ω*, *Z* and *c*) which affect final population size but, for reasons described above, have no impact on whether or not a population is viable.

### The impact of insecticide deployment prior to the emergence of resistance

**Fig. 3:**
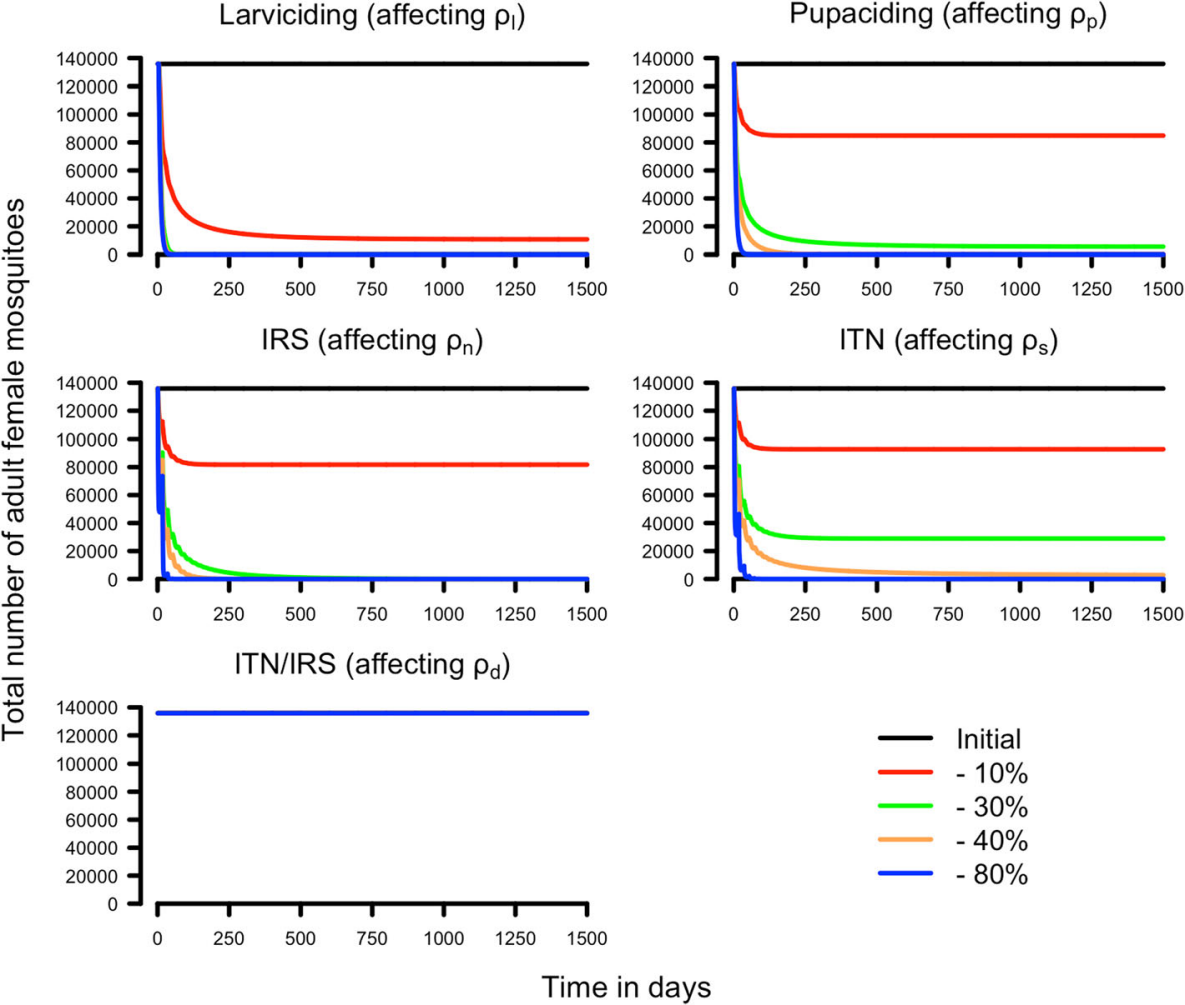
Simulations of the impact of insecticidal interventions on the female adult population size. Interventions were simulated by the decreasing survival that would plausibly occur at five different stages: larval (*ρ*_*l*_), pupal (*ρ*_*p*_), adult females host-seeking (*ρ*_*s*_), adult females resting (*ρ*_*n*_) and adult males (*ρ*_*d*_). The legend shows the percentage of decreased survival imposed on each parameter i.e. 0 (the initial group) -10, -30, -40 and -80%. *Abbreviations*: IRS, indoor residual spraying; ITN, insecticide-treated net

**Fig. 4:**
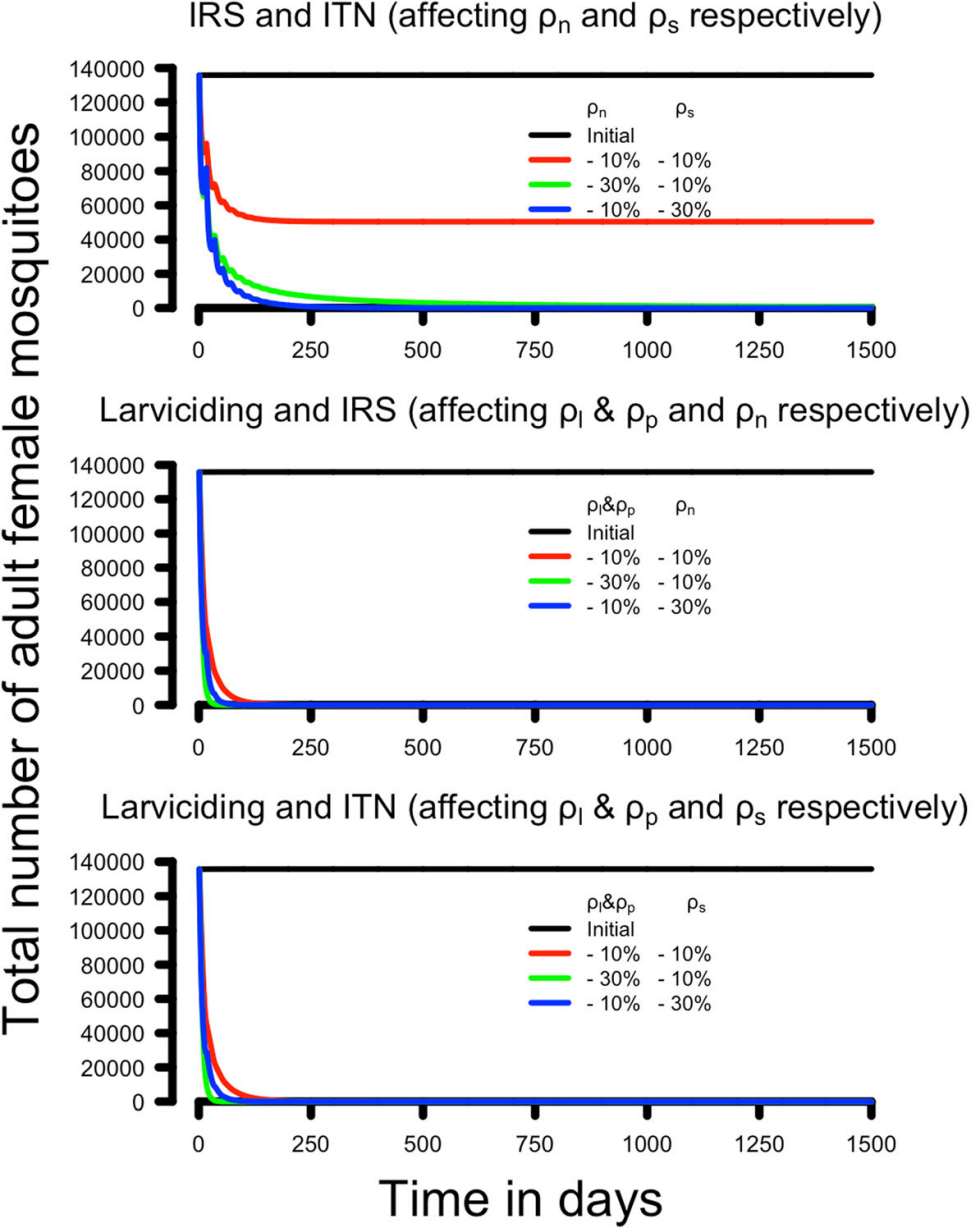
Simulations of the impact of combined insecticidal interventions on the female adult population size. Interventions were simulated by decreasing the survival that would plausibly occur in three combined interventions. The legend on each panel shows the percentage of decreased survival imposed on each parameter by the intervention. IRS combined with ITNs reduces female survival while resting (*ρ*_*n*_) and host-seeking (*ρ*_*s*_). Larviciding combined with IRS reduces survival of both sexes in the larvae and pupal stages (*ρ*_*l*_ and *ρ*_*p*_ ) and in adult females resting stages (*ρ*_*n*_). Larviciding combined with ITNs reduces survival of both sexes in the larvae and pupal stages (*ρ*_*l*_ and *ρ*_*p*_ ) and adult females while host-seeking (*ρ*_*n*_). *Abbreviations*: IRS, indoor residual spraying; ITN, insecticide-treated net

**Fig. 5:**
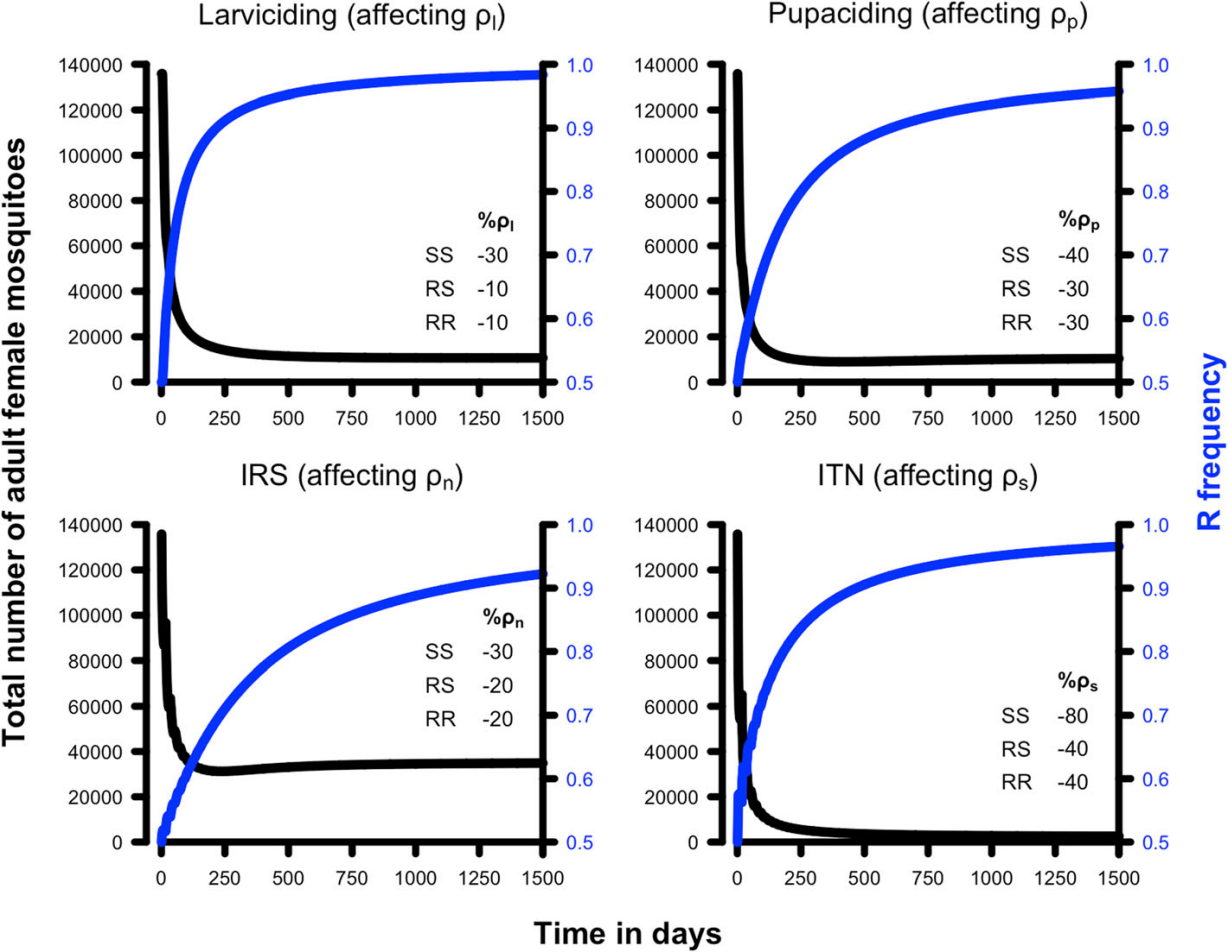
The potential impact of resistance on single-insecticide control interventions. The legend on each panel gives the percentage of decreased survival caused by the intervention for each genotype. The blue line shows the resistant allele frequency over time and the black line shows the number of female adult mosquitoes. The rapid decline in adult population size post-intervention shows that the magnitude of the resistance phenotype was not sufficient to prevent a population crash (although the smaller, resistant population were sufficient to allow malaria transmission; see Table 5 and main text for details). Resistance was assumed to be dominant, interventions started with a resistance allele frequency of 50% and the three genotypes in Hardy-Weinberg equilibrium. *Abbreviations*: IRS, indoor residual spraying; ITN, insecticide-treated net; SS, the homozygous sensitive genotype; SR, the heterozygous sensitive/resistant genotype; RR, the homozygous resistant genotype

**Fig. 6:**
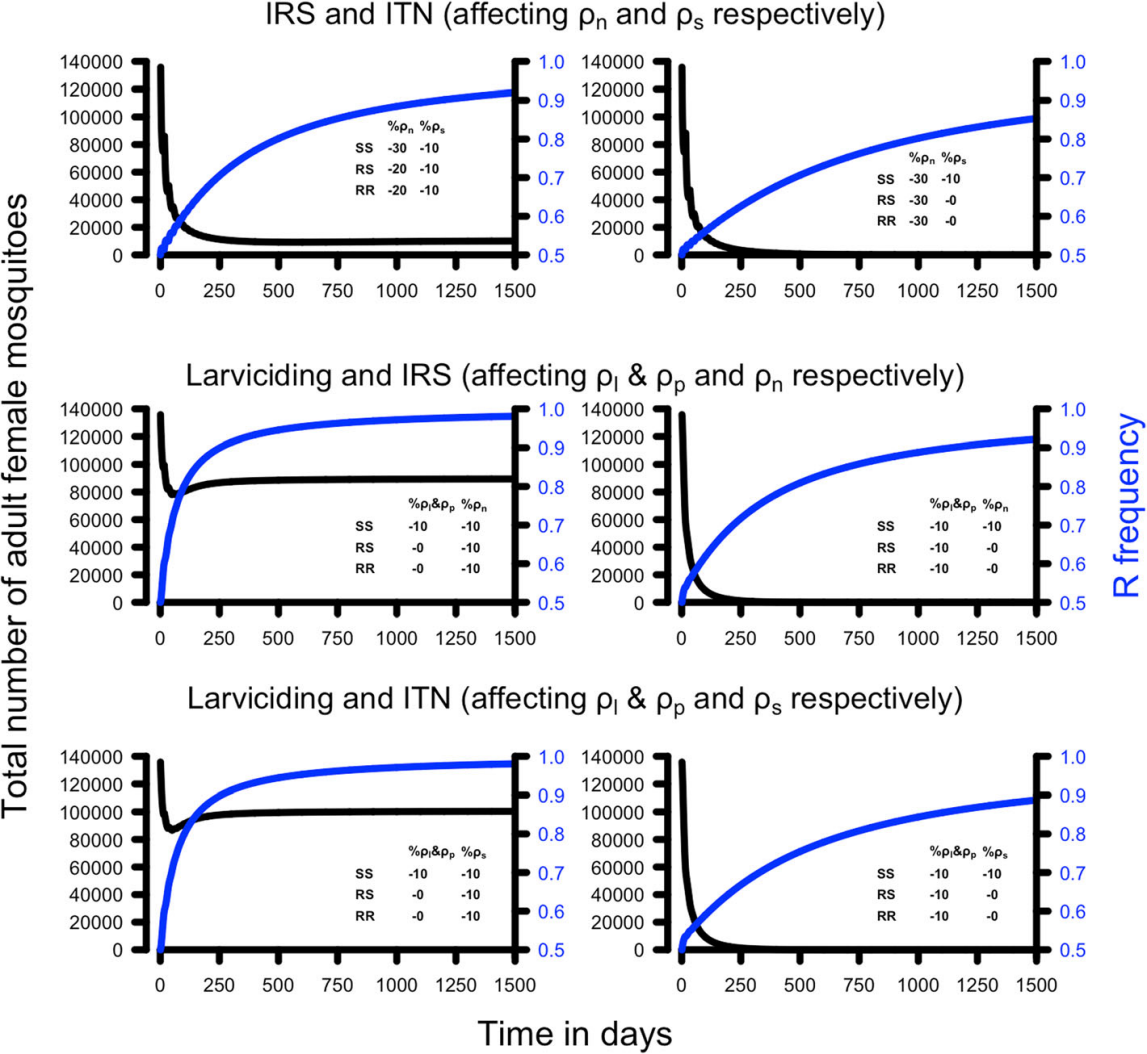
The potential impact of insecticide resistance on combined-insecticide control interventions. The legend in each panel shows the percentage of decreased survival imposed by the intervention on each parameter for each of the three genotypes. The blue line shows the resistant allele frequency over time and the black line shows the total number of female adult mosquitoes. As in Fig. 5, the magnitude of the resistance phenotype was not sufficient to prevent a population crash although the smaller, resistant, populations may be sufficiently large to allow malaria transmission (see Table 6 and main text for details). Resistance was assumed to be dominant, interventions started with a resistance allele frequency of 50% and the three genotypes in Hardy-Weinberg equilibrium. *Abbreviations*: IRS, indoor residual spraying; ITN, insecticide-treated net; SS, the homozygous sensitive genotype; SR, the heterozygous sensitive/resistant genotype; RR, the homozygous resistant genotype

An equilibrium adult female population size (*A*_*f*_(*t*)) of 135,878 was reached using the default parameterization given in Table 1; the ability of the controlled population to transmit disease can be investigated using a Ross-Macdonald model calibrated as described in Table 2. This equilibrium population size of adult females served as the baseline adult female population size in the absence of intervention in all simulations/scenarios (i.e. Figs. 3, 4, 5 and 6).

**Table 2:** Parameters used in the Ross-Macdonald transmission calculations

Symbol	Meaning	Default value
m	Mosquito density, i.e. number of adult female mosquitoes per human host	110
a	Mosquito biting rate on humans	0.25
*b*_1_	Probability a bite from an infectious mosquito transmits malaria to susceptible humans	0.5
*b*_2_	Probability a susceptible mosquito acquires a malaria infection when biting an infectious human	0.15
r	Rate at which humans recover from a malaria infection	0.01

Note that mosquito density, *m*, is a relatively large number of adult females per host and thus represents an area of high malaria transmission; see Additional file 3 for more information

We investigated the likely impact of single-insecticide interventions by assuming insecticide deployment decreases survival probabilities in various parts of the mosquitoes’ lifestyle by 10, 30, 40 or 80% (Table 3). Whether these impacts are sufficient to interrupt malaria transmission can be investigated using Eq. 22 with the calibration developed above (as summarised in Tables 1 and 2) to identify the threshold density of mosquitoes below which malaria transmission cannot be sustained. The equilibrium number of females present after the intervention are given in Table 3. A 30% reduction of the larval daily survival (0.94 to 0.66) resulted in extinction of the mosquito population, and hence interruption of malaria transmission. The pupae survival probability, *ρ*_*p*_, would have to be lowered by 40% (0.55 to 0.33) to drive the population to extinction. Note that we modelled pupae independently from larvae, because pupae do not feed and therefore are believed not to incur density-dependence regulation, but in practice both stages share the same physical space and interventions such as larviciding may affect both stages. This scenario of decreasing survival of only the pupal stage is, therefore, very unlikely to be used in the field. However, it serves to show that theoretically we would have to reduce pupae daily survival more than larval survival to achieve the same level of reduction in the adult female population, the underlying reason being that the pupal stage is shorter so daily survival must be much lower to achieve comparable overall killing to the longer larval stage. Targeting adult females only in the non-feeding, resting stage, *ρ*_*n*_, requires a more modest decrease of 30% (0.96 to 0.67) to generate near-extinction of the mosquito population with consequent cessation of malaria transmission. ITNs target adult females seeking a host for a blood meal and is one of the most widespread malaria interventions; our results suggest it would be necessary to decrease survival during the seeking stage, *ρ*_*s*_, by around 40% (0.96 to 0.58) to eliminate the mosquito population.

**Table 3:** The impact of insecticide-based interventions that target one stage of the life-cycle

Reduction in parameter value	Intervention [parameter affected]			
	Larviciding [*ρ*_*l*_]	Pupacide [*ρ*_*p*_]	IRS [*ρ*_*n*_]	ITN [*ρ*_*s*_]
-10%	10,711 (877)	84,873 (877)	81,717 (3803)	92,760 (1328)
-30%	0 (877)	5483 (877)	0 (71,767)	28,909 (3163)
-40%	0 (877)	0 (877)	0 (373,954)	2502 (5113)
-80%	0 (877)	0 (877)	0 (~10^10^)	0 (97,845)

The table gives the equilibrium adult female population sizes achieved after the interventions plotted on Fig. 3. The associated critical values of the adult female population size below which malaria transmission will cease, is given in parenthesis (M’, obtained from Eq. 22); note that the parameters *ρ*_*l*_ and *ρ*_*p*_ do not affect adult mosquito mortality so the target population size is unaffected by their value). Note that male adult survival *ρ*_*d*_ has no impact on female population size so is not included in this analysis

Figure 3 illustrates the dynamics of these interventions summarised in Table 3, taking the pre-intervention population of 135,878 as its starting point. The bottom panel of Fig. 3 shows the impact of reducing adult male survival. As expected, it is not possible to decrease the female adult population by targeting the male population alone because our model assumes males can mate multiple times and so changes in male number caused by reduced *ρ*_*d*_ have no impact on the size of the next generation unless they are so large as to eliminate all males. We use a similar approach to investigate the impact of combined-insecticide interventions by assuming the insecticides reduced life-cycle survivals by 10 or 30%. The results are summarised in Table 4. The hypothetical example of combining IRS and ITNs is sufficient to drive the mosquito population to extinction or to a very small size that is well below the threshold for interruption of malaria transmission assuming a decrease in survival of 10% in the non-host-seeking females (*ρ*_*n*_ = 0.87) and 30% in the host-seeking females (*ρ*_*s*_ = 0.5), or *vice versa* (Table 4). Combining larviciding with either IRS or ITN suggests that small reductions (10%) in both parameters are sufficient to render the mosquito population inviable and to interrupt malaria transmission (Table 4).

**Table 4:** The impact of insecticide-based interventions that target two stages of the life-cycle

Reductions in parameter values	Intervention [parameters affected]		
	IRS and ITN [*ρ*_*n*_ and *ρ*_*s*_]	Larviciding and IRS [*ρ*_*l*_, *ρ*_*p*_ and *ρ*_*n*_]	Larviciding and ITN [*ρ*_*l*_, *ρ*_*p*_ and *ρ*_*s*_]
-10% and -10%	50,432 (5329)	0 (3803)	0 (1328)
-30% and -10%	0 (94,683)	0 (3803)	0 (1328)
-10% and -30%	0 (11,307)	0 (71,767)	0 (3163)

The table gives the equilibrium adult female population sizes achieved after the interventions plotted on Fig. 4 with, in parenthesis, the critical value of adult female population size below which malaria transmission will cease

The dynamics of the interventions shown in Figs. 3 and 4 suggest that interventions of this magnitude may have a rapid effect acting on a timescale of weeks. Note, however, that our simulations assumed instantaneous deployment of the insecticide-based interventions and so illustrate its fastest possible impact on the local mosquito population. In reality, an intervention may take days, weeks or even months to deploy and in this case the reduction in population size will be much slower. Importantly, the final equilibrium population size will not be affected by how rapidly the intervention is deployed and the proportionate reduction in population size can be obtained from Table 3 noting that the original population size was 135,878 (so, for example, Table 3 shows that if larviciding decreases larval survival by 10%, this will reduce the population size to 10,711 which is a 92% reduction in population size).

### The impact of resistance on insecticide-based interventions

The simulations shown in Figs. 3 and 4 assumed only a single genotype was present, i.e. the homozygous sensitive, SS, genotype. We introduced resistance SR and RR genotypes and re-ran these simulations to illustrate the potential impact of resistance on insecticide-based control programmes. The resistant allele, R, was assumed to be present at a frequency of 50% and was assumed to be dominant. It is important to note, given our simplifying assumption that no fitness cost is associated with resistance, that if resistance spreads from a starting frequency of 50% it will spread from any starting frequency, including very low ones. Consequently, our results and conclusions are unaffected by choice of initial resistance frequency. The reason we chose a starting frequency of 50% was to emphasise how rapidly resistance spreads and potentially undermines control, once it reaches detectable frequencies (if we start with lower initial frequencies, or recessive gene action, then there is a long period before resistance reaches significant frequencies). We start with the equilibrium population size that was obtained under the default parameters (i.e. 135,878 adult females) then impose interventions that have illustrative, differential effects on the sensitive and resistant genotypes (as defined in the panels of Figs. 5 and 6).

Examples of hypothetical single-insecticide interventions are shown in Fig. 5. In all cases, resistance spread rapidly during the intervention. However, the magnitude of the resistance phenotype was insufficient to prevent the mosquito population from rapid, large and sustained reductions post-intervention. Despite this apparent success, Table 5 suggests this “crashed” population was sufficiently large that malaria transmission would be maintained. The adult population sizes in the absence of resistance (i.e. if only SS genotypes were present) would be zero in each example (second column of Table 5, but the presence of resistance may allow a viable mosquito population to be maintained once resistance has been fixed (fourth column of Table 5) that is sufficiently large so that malaria transmission is possible.

**Table 5:** The impact of resistance on control interventions that target a single stage of the life-cycle. This is quantified by adult mosquito population size

Intervention [parameter]	Adult population size; SS genotypes	Resistance calibration	Adult population size; RR genotypes	Population re-established?	Transmission restarts? (R_0_)
Larviciding [*ρ*_*l*_]	0 (877)	Top left panel of Fig. 5	10,711 (877)	Yes	Yes (12)
Pupacide [*ρ*_*p*_]	0 (877)	Top right panel of Fig. 5	5483 (877)	Yes	Yes (6.3)
IRS [*ρ*_*n*_]	0 (71,767)	Lower left panel of Fig. 5	29,636 (15,946)	Yes	Yes (1.8)
ITN [*ρ*_*s*_]	0 (97,845)	Lower right panel of Fig. 5	2502 (5113)	Yes	No (< 1)

The column “Adult population size; SS genotypes” shows the equilibrium female mosquito population sizes post-intervention assuming no resistance is present (i.e. only SS genotypes) and, in brackets, the critical adult female population size required to block malaria transmission using the Ross-Macdonald approach (i.e. M’, obtained from Eq. 22); all these interventions eradicated the local mosquito population and hence stopped malaria transmission. The column “Adult population size; RR genotypes” gives the equivalent values of population size and M’ but assuming only RR genotypes are present. The parameterisations are as shown in the panel captions of Fig. 5; for example, the first row of this Table is equivalent to the top left panel of Fig. 5, i.e. the values are calibrated by assuming that the insecticide intervention reduces larval survival *ρ*_*l*_ of the SS genotype by 30% while the RR and RS genotypes are less affected by insecticide and their larval survival during the intervention is 10% lower than in the absence of insecticide. Comparison of the “SS genotypes” with the “RR genotypes” column reveals whether the spread of resistance during the interventions shown in Fig. 5 is sufficient for mosquito populations to become re-established (we define this as a greater than 100-fold increase in adult female numbers) and, if so, whether they recover to the extent that malaria transmission restarts. If transmission does restart, we give estimated R_0_ (from Eq. 19) to quantify the magnitude of this resurgence (baseline before control, was R_0_ = 155)

The analogous example of combined-insecticide interventions in the presence of resistance is shown in Fig. 6 and summarised in Table 6. The same basic dynamics occurred as for single-insecticide interventions, i.e. a rapid increase in resistance and an immediate fall in the adult female population. The impact of the latter was more heterogeneous. All interventions would have reduced mosquito populations to negligible sizes and blocked transmission (column 2 of Table 6). However, the spread of resistance allowed mosquito population sizes to recover sufficiently that disease transmission would re-start in 2 of the 6 scenarios (columns 4 to 6 of Table 6).

**Table 6:** The impact of resistance on control interventions that target two or more stages of the life-cycle

Intervention [parameters]	Adult population size; SS genotypes	Resistance calibration	Adult population size; RR genotypes	Population re-established?	Transmission restarts? (R_0_)
ITN and IRS [*ρ*_*s*_and *ρ*_*n*_]	0 (94,683)	Top left panel of Fig. 6	8517 (21,515)	Yes	No (< 1)
		Top right panel of Fig. 6	0 (71,767)	No	No (< 1)
Larviciding and IRS [*ρ*_*l*_ & *ρ*_*p*_and *ρ*_*n*_]	0 (3803)	Middle left panel of Fig. 6	81,717 (3803)	Yes	Yes (21)
		Middle right panel of Fig. 6	0 (877)	No	No (< 1)
Larviciding and ITN [*ρ*_*l*_ & *ρ*_*p*_ and *ρ*_*s*_]	0 (1328)	Lower left panel of Fig. 6	92,760 (1328)	Yes	Yes (70)
		Lower right panel of Fig. 6	0 (877)	No	No (< 1)

The parameterisations are as shown in the panel captions of Fig. 6. See caption in Table 5 for further details

## Discussion

Insecticides are used in many contexts to reduce insect-borne disease transmission. We have combined mosquito demographics, genetics and malaria epidemiology to provide a methodology to simultaneously investigate the impacts of insecticide deployments in reducing or preventing the transmission of infections and the threat posed by resistance. To our knowledge, this synthesis has not been attempted prior to this study although many previous studies have addressed individual aspects of these requirements (space precludes a detailed discussion of this previous work but access to the modelling literature can be obtained, for example, from [16, 19, 21, 24, 25, 35, 41] and recent reviews such as [42]). We have taken a standard demographic model and added three novel factors: the ability to track insecticide resistance spread within the mosquito demography, derived an equation for R_0_ of mosquitoes that predicts whether interventions will drive local mosquitoes populations to extinction, and finally used the parameters of insect demography to derive a Ross-Macdonald equation for R_0_ for malaria that indicates whether it is likely that the reduction in mosquito numbers and/or their longevity is sufficient to interrupt malaria transmission. In summary, we have described a transparent methodology that allows researchers to investigate specific scenarios, while being sufficiently flexible that the genetic component can also be used to investigate other systems such as sex-linked resistance and genetic control mechanisms [18].

We developed a basal model as proof-of principle which is sufficiently flexible to allow alternative control strategies to be incorporated and evaluated. One such example is the proposal to target male mating swarms to reduce mosquito population size [43]. We assumed above that males could effectively inseminate an infinite number of females, hence, the number of males made no difference to the population size (and hence male survival was immaterial; lower panel of Fig. 3). This is a simplifying assumption, often made in ecology/demography, that recognises that female number is the usual determinant of population size. We could relax this assumption. For example, if males are believed to be unable to inseminate more than ten females per night, we could restrict the number of mated females per night to less than ten times the adult male population size. Similarly, for species where mating occurs in a male swarm (such as *An. gambiae*) if a male population size is reduced to the extent that females find it difficult to locate a swarm then the female mating probability can be reduced. We ignored these potential complications in this manuscript to focus on the basic genetics and demography but note that they can be included in modelling directed at more specific intervention scenarios. Similarly, we assume a single genetic locus encodes resistance but the methodology could be extended, albeit with a substantial increase in complexity, to include two genetic loci which would allow users to investigate the impact of joint-insecticide strategies such as the use of mixtures (e.g. [18, 20, 44]). This assumption that one gene encodes resistance to an insecticide has been commonly made throughout the literature (e.g. [45–49] and subsequent work). The results presented herein are also valid if resistance is coded by polygenes (i.e. resistance level is modulated by a large number of genes, each with a very small effect). For example, Tables 5 and 6 and Figs. 5 and 6 show the impact of a reduction in mortality on mosquito population size and disease transmission caused by IR. The genetic basis of the degree of IR is immaterial for this impact, e.g. a reduction in larval mortality by 10% has the same impact irrespective of whether its genetic basis is a single gene or many genes. The dynamics of spread will be very different between single- and poly-genetic resistance [50] but the impact of resistance on control can be investigated in the same way. A final, strategic application is to simulate interventions, quantify how rapidly resistance spreads, and use these dynamics to extract the selective advantage of resistance which is a key input parameter for calibrating genetic models of IR evolution.

Operationally, ITNs and IRS may have two additional effects not captured in our model: repelling and possibly diverting mosquitoes to alternative hosts due to insecticide irritation (e.g. [19]) and/or the physical barrier of the net, and lengthening the duration of the gonotrophic cycle leading to a reduced oviposition rate [24]. The methodology can also incorporate behavioural changes that may evolve in response to insecticide resistance [51–54], for example, a reduced tendency to rest indoors after feeding, which will lower mortality rates during the female gonadotrophic cycle. These factors can be brought into insecticide resistance modelling but we have ignored these possible effects for simplicity; in particular, a formal sensitivity analysis (discussed later) would reveal the extent to which behavioural changes may affect the evolution of resistance and its impact on disease transmission.

The Ross-Macdonald (R-M) approach is the easiest algebraic method of predicting whether disease transmission will cease (see [32] for an extensive review of R-M). It is also flexible: for example, we assume a female always finds a mate on the first day of emergence, and that the adult female feeding cycle is as quantified as in Eq. 21, but heterogeneity in such factors can be regarded as occurring in different mosquito ‘species’ and the overall R_0_ calculated from the relative frequencies of these different ‘species’. The two main criticisms of R-M, that it does not allow super-infection or acquired human immunity, do not apply in our usage because cessation of malaria transmission at R_0_ < 1 implies no infections and hence no super-infection, and no acquired immunity. The drawback of R-M is that it generates a simple yes/no prediction of whether the mosquito population has the capacity to sustain malaria transmission, but it is not a robust method to quantitatively predict the intensity of malaria transmission nor its epidemiological impact; the latter depends on factors such as malaria super-infection in humans, levels of human acquired immunity, malaria importation rates and so on [32]. The methodology developed here is focused on mosquito demography and, if malaria transmission is identified as being viable, then these details of mosquito demography need to be passed to more sophisticated, individual-based simulations models of malaria transmission that do incorporate the human elements of malaria epidemiology (e.g. [55, 56]) to simulate the impact on human populations.

The results of the interventions targeting single stages of the mosquitoes’ life-cycle (using the PRCC values in Fig. 2 and examples in Fig. 3) indicate that the most effective method of controlling the mosquito population, all other factors being equal, would be to target the larval and the adult resting stages. These results reflect the belief that larval survival has a great impact on the adult population density although, as pointed out by White et al. [24], it does not kill adult mosquitoes that are potentially infectious so may have a smaller impact on disease transmission (i.e. female adult death rate is not affected). Alternatively, it may be better to target the host-seeking female mosquitoes to reduce disease transmission; this may make little difference to overall mosquito population size but their reduced longevity makes a substantial difference to malaria transmission. The strategy with the most impact will also depend on individual species demography and local environmental conditions. In our parameterisation, larvae were a good intervention target because they spend ten days in this stage so mortality at this stage operates over ten days. Conversely, we assume a ten day extrinsic incubation period (EIP) so mortality in resting females operates over nine days (Eq. 21). However, if temperature falls such that the EIP increases to 20 days then female mortality while resting will operate over 18 days and this stage may become a far more effective point of control. In reality “all other factors” are not equal as there are operational and financial differences associated with each strategy. An obvious example from laviciding is how to identify a substantial proportion of the breeding sites (e.g. [57]) because these depend on local mosquito ecology that may vary widely even within a species. Despite this requirement to identify breeding sites, larviding is likely to become increasing important as the most plausible insecticide-based method of targeting the outdoor-biting mosquito species responsible for “residual” malaria transmission once the primary indoor resting/biting species have been controlled. Our methodology is therefore capable of providing insight into how control may be optimised by balancing operational difficulty against likely impact; for example, contrasting a low impact, operationally simple and hence widespread intervention, against an operationally complex, more focussed approach with high local impact on mosquito populations. We emphasise that this manuscript primarily describes methodological advances, tying together the separate strands of insecticide deployment, insect demography and bionomics, the evolution of resistance and the impact of resistance on disease transmission. The conclusions described above, for example the high impact of larviciding, are correct for the specific instances we investigated but could not yet be used as a basis for general policy recommendations. Such recommendations would need to be based on a far more detailed sensitivity analysis than the rather arbitrary one used here (Additional file 3). Full exploration of plausible parameter space may well conclude that no one strategy is universally superior, but that the optimal strategy depends on local conditions (as occurred, for example, in our recent work on whether insecticides should be deployed sequentially or in mixtures [18]).

There is increasing emphasis on the need for rational, co-ordinated efforts to control disease vectors, and integrated vector management (IVM) schemes are now an integral part of WHO policy [58]. Achieving the goals of programmes such as Roll Back Malaria may require an integrated approach combining disease treatment and interventions against both adult and larval stages of the vector [25]. IVM strategies often deploy combinations of interventions targeting two or more stages of the life-cycle. Combinations are intuitively likely to be more effective than interventions targeting a single stage. In reality, there are a number of important confounding factors that can affect the effectiveness of combined insecticide interventions. A comprehensive review of these factors can be found in [59] but they include, for example, (i) whether the insecticides act independently or may interfere or synergise with each other, (ii) whether the durations of insecticide persistence are matched or whether one decays more rapidly leaving the other to act alone for extended periods, and (iii) the behaviour of the vector, such as the extent to which it is anthropophilic and/or endophilic. As a real example, data from the Solomon Islands [60] suggested that house spraying (with DDT) was more effective than ITNs but that the amount of the insecticide required would be reduced if ITNs were also used. However, the same study was not able to associate reduction in malaria cases with larviciding (with temephos) in combination with other interventions. In particular, the use of IRS and ITNs in combination is thought to increase the probability of a mosquito meeting an insecticide, and help to reach and maintain high coverage levels that are often difficult to attain with single deployment strategies [61, 62]. Similarly, the addition of larviciding to ITN deployment has been shown to be highly beneficial [63] as has larval source management, although this depends on the ability to identify a large proportion of breeding sites [64]. The problem is that the more effective an intervention, the greater the selection for resistance; trading short-term benefits in reducing disease transmission, against longer-term impacts of driving IR means that both processes should ideally be combined in the same model as was done here.

Resistance is a constant threat to interventions and our results suggest that when deploying a single intervention, even a small increase in survival due to insecticide resistance may be sufficient to restore a mosquito population to sustainable levels (Tables 5 and 6). The results presented above suggest that, in terms of reducing adult female population size, the use of larviciding seems an effective option either alone or in combination, although unlike ITN and IRS, it will not reduce the longevity of adult females. Importantly, it is likely that resistance will spread faster if insecticides target the larval stages rather than the adult stages. This occurs for two reasons. First, because insecticides have a bigger impact on larval survival: their effects are compounded over the ten days of larval life, so selection for resistance may be higher. Secondly, because larviciding applies selection pressure on both sexes; in contrast, adulticides used in IRS or ITNs differentially target females, leaving the exophilic males as a sort of unexposed refugia shielded from selection pressures [19, 65].

There are frequent calls to ‘model resistance’ (e.g. [66]) and our modelling approach describes the parameters required to fully calibrate the system, which constitutes a type of ‘shopping list’ of variables that should be collected in the field. It is important to note that we are not attempting here to investigate and evaluate specific insecticide-based interventions, but are concentrating on developing the methodology by which this may be done. The main impediment to investigating specific interventions is that many of the required parameter values are largely unknown. As a specific example, the number of male mosquitoes entering homes (and hence potentially encountering insecticides on wall and ITNs) is often unknown because many researchers simply discard males from their collections as they play no role in malaria transmission. We therefore recognise that accurate calibration of individual ecological/epidemiological settings is currently impossible. We have focused on developing the model, obtaining preliminary, illustrative results, and anticipate that its main use will be in future sensitivity analyses. These analyses recognise that accurate calibration is often impossible and instead explore a single, plausible parameter space (e.g. [18]); the key operational issue then is to identify what interventions are best (however ‘best’ is defined, e.g. cost, simplicity, short- or long-term impact on transmission) and whether the ‘best’ policy depends on local vector bionomics and patterns of transmission. Our results are, therefore, preliminary and serve the purpose of demonstrating the potential of our computational approach. If one policy always performs better irrespective of underlying parameters, then it is a robust conclusion to use that policy. If some policies work better in certain situations and worse in others, then analysis of the models can show under which conditions (i.e. parameter combinations) each policy works best (most obviously using classification trees, e.g. [67]) and hence identify policies appropriate to local conditions. The illustrative analyses we performed explored the comparative impact of ITNs, IRS and larvicides, and quantified the benefits that can be achieved by combining these interventions.

## Conclusions

We develop and describe a stand-alone model that simultaneously incorporates mosquito demography and the genetics of resistance, to simulate the impact on disease transmission and the extent to which this impact is threatened by the spread of resistance. Future development would be to link the model to one of health economics to investigate the cost-effectiveness of each intervention and the extent to which short-term gains in control might be offset by longer-term losses due to resistance. There is currently intense interest in modelling malaria to underpin elimination efforts [66], and models such as the one developed here linking demography and the genetics of resistance have a key role to play in designing sustainable control and elimination strategies.

## Additional files

Additional file 1: A mathematically-rigorous derivation of R_0_ for anophelene mosquitoes. (PDF 141 kb)

Additional file 2: An equivalent, intuitive, derivation of R_0_ for anophelene mosquitoes. (DOCX 18 kb)

Additional file 3: Notes on model calibration. (DOCX 21 kb)

## Abbreviations

DDPR: Density-dependent population regulation
EIP: Extrinsic incubation period
IR: Insecticide resistance
IRS: Indoor residual spraying
ITN: Insecticide-treated net
IVM: Integrated vector management
R_0_: Basic reproductive number
RR: The homozygous resistant genotype
SR: The heterozygous sensitive/resistant genotype
SS: The homozygous sensitive genotype
