# Supplementary material for: Modelling the impact of insecticide-based control interventions on the evolution of insecticide resistance and disease transmission
Source: Parasit Vectors. 2018 Aug 28;11:482. doi: 10.1186/s13071-018-3025-z (PMC6114906; doi:10.1186/s13071-018-3025-z)
Supplement: Supplementary file 1 — A mathematically-rigorous derivation of R0 for anophelene mosquitoes. (PDF 141 kb) [file 13071_2018_3025_MOESM1_ESM.pdf]

Modelling the impact of insecticide-based control interventions on the evolution of insecticide resistance and disease transmission
Supporting Information: Additional File 1

Susana Barbosa, Katherine Kay, Nakul Chitnis, Ian M. Hastings

Here we derive the basic reproductive number for the model of female mosquito population dynamics ignoring differences in genotypes. We ignore male mosquitoes because we assume that female mosquitoes can always find a mate. In contrast to the main text, we omit the superscripts and use the index i to refer to the number of mosquitoes at the beginning of the day to make the following calculations easier. The model for female mosquitoes for one genotype can be written as

$$x_1(t+1) = \varphi\beta\rho_n y_\tau(t), \tag{1a}$$
$$x_i(t+1) = \rho_e x_{i-1}(t) \quad \text{for } 2 \leq i \leq \theta_e + 1, \tag{1b}$$
$$x_i(t+1) = \frac{\rho_l}{1 + \frac{c_{i-1}}{Z}\left(\sum_{k=\theta_e+1}^{\theta_e+\theta_l} \omega_k x_k(t)\right)} x_{i-1}(t) \quad \text{for } \theta_e + 2 \leq i \leq \theta_e + \theta_l + 1, \tag{1c}$$
$$x_i(t+1) = \rho_p x_{i-1}(t) \quad \text{for } \theta_e + \theta_l + 2 \leq i \leq \zeta, \tag{1d}$$
$$y_1(t+1) = \rho_p x_\zeta(t) + \rho_s(1-H)y_1(t) + \rho_n y_\tau(t), \tag{1e}$$
$$y_2(t+1) = \rho_s H y_1(t), \tag{1f}$$
$$y_i(t+1) = \rho_n y_{i-1}(t) \quad \text{for } 3 \leq i \leq \tau. \tag{1g}$$

If $\tau = 2$, then there is no (1g) while the other subequations remain the same. If $\tau = 1$, then, (1a) becomes,

$$x_1(t+1) = \varphi\beta\rho_s H y_1(t), \tag{2a}$$

and (1e), (1f) and (1g) combine to provide,

$$y_1(t+1) = \rho_p x_\zeta(t) + \rho_s y_1(t), \tag{2b}$$

while all other subequations remain the same.

For notational convenience, we define $\eta = \zeta + \tau$, and denote the non-negative orthant of $\mathbb{R}^\eta$ by $\bar{\mathbb{R}}^\eta_+$. The system can be written in the form,

$$\mathbf{x}(t+1) = A(\mathbf{x}(t))\mathbf{x}(t), \tag{3}$$

where $\mathbf{x}(t) \in \bar{\mathbb{R}}^\eta_+$, $A(\mathbf{x})$ is the $\eta \times \eta$ projection matrix and all elements of $A(\mathbf{x})$ are continuously differentiable on an open domain $\mathcal{D} \subset \mathbb{R}^\eta$ with $\bar{\mathbb{R}}^\eta_+ \subset \mathcal{D}$. For initial conditions in $\bar{\mathbb{R}}^\eta_+$, the system of equations (1) has a unique solution that remains in $\bar{\mathbb{R}}^\eta_+$ for all time.

The extinction equilibrium of (1) is given by,

$$\mathbf{x}_e = \mathbf{0}, \tag{4}$$

where $\mathbf{0}$ is defined as the origin in $\mathbb{R}^\eta$.

We let $A(\mathbf{x}) = F(\mathbf{x}) + T(\mathbf{x})$ where $F(\mathbf{x})$ is the fertility matrix and $T(\mathbf{x})$ is the transition matrix. The element $F_{i,j}(\mathbf{x})$ is the number of new mosquitoes born in stage i per mosquito in stage j per time step. The element $T_{i,j}(\mathbf{x})$ is the proportion of mosquitoes in stage j that survive one time step to enter stage i.

The basic reproductive number, R_0, is the expected number of female offspring of one female mosquito through her life span, in the absence of density dependence. We use the methods from Cushing (2009) [**?**] to define the basic reproductive number of the mosquito population as,

$$R_0 = \sigma\left(F(\mathbf{0})(I - T(\mathbf{0}))^{-1}\right), \tag{5}$$

where I is the $\eta \times \eta$ identity matrix and $\sigma(M)$ is the spectral radius of M (the maximum absolute value of the eigenvalues of M).

We derive R_0 for $\tau = 2$ and $\theta_p \geq 2$. Although the details are different, the calculations are similar for $\theta_p = 1$ and $\tau \geq 3$. Calculations for $\tau \geq 3$ provide exactly the same expression for R_0, while calculations for $\theta_p = 1$ provides a slightly different formulation as described below. The matrices $F(\mathbf{0})$ and $T(\mathbf{0})$ have the form,

$$F = \begin{pmatrix} 0 & \cdots & 0 & F_{1,\eta} \\ 0 & \cdots & 0 & 0 \\ \vdots & \ddots & \vdots & \vdots \\ 0 & \cdots & 0 & 0 \end{pmatrix}, \tag{6}$$

$$T = \begin{pmatrix} 0 & \cdots & 0 & 0 & 0 \\ T_{2,1} & \ddots & \vdots & \vdots & \vdots \\ 0 & \ddots & 0 & 0 & 0 \\ \vdots & \ddots & T_{\eta-1,\eta-2} & T_{\eta-1,\eta-1} & T_{\eta-1,\eta} \\ 0 & \cdots & 0 & T_{\eta,\eta-1} & 0 \end{pmatrix}. \tag{7}$$

The only nonzero element of $F(\mathbf{0})$ is in its first row and last column. The only nonzero elements of $T(\mathbf{0})$ are its subdiagonal and the two elements in the second-last row and the second-last and last columns. Since $F(\mathbf{0})$ only has a nonzero element in its first row, $F(\mathbf{0})(I - T(\mathbf{0}))^{-1}$ has nonzero elements only in its first row. The largest eigenvalue of $F(\mathbf{0})(I - T(\mathbf{0}))^{-1}$ is therefore the first element of its diagonal which is the product of $F_{1,\eta}(\mathbf{0})$ and the element in the ηth row and first column of $(I - T(\mathbf{0}))^{-1}$.

The determinant, D, of $(I - T(\mathbf{0}))$ is given by,

$$D = 1 - T_{\eta-1,\eta-1}(\mathbf{0}) - T_{\eta-1,\eta}(\mathbf{0})T_{\eta,\eta-1}(\mathbf{0}). \tag{8}$$

The $(\eta, 1)$ element of $(I - T(\mathbf{0}))^{-1}$ is the product of all elements of the subdiagonal of $T(\mathbf{0})$ divided by the determinant of $I - T(\mathbf{0})$,

$$\frac{\prod_{k=1}^{\eta-1} T_{k+1,k}(\mathbf{0})}{1 - T_{\eta-1,\eta-1}(\mathbf{0}) - T_{\eta-1,\eta}(\mathbf{0})T_{\eta,\eta-1}(\mathbf{0})}.$$

Substituting in the expressions for $F(\mathbf{0})$ and $T(\mathbf{0})$ provides,

$$R_0 = \frac{\varphi\beta\rho_e^{\theta_e}\rho_l^{\theta_l}\rho_p^{\theta_p}\rho_s H\rho_n^{\tau-1}}{1 - \rho_s(1 - H) - \rho_s H\rho_n^{\tau-1}}, \tag{9}$$

which is the expected number of female offspring of one female mosquito through her life in the absence of density dependence. The numerator is the product of the number of female eggs laid per oviposit, the probability of surviving the egg stage, the density-independent probability of survival the larval stage, the probability of surviving the pupal stage, the probability of successfully

mating, and the probability of surviving one feeding cycle. The reciprocal of the denominator is the expected number of feeding cycles that one mosquito will have in her life span.

Similar calculations for $\tau = 1$ provide,

$$R_0 = \frac{\varphi\beta\rho_e^{\theta_e}\rho_{l0}^{\theta_l}\rho_p^{\theta_p}\rho_s H}{1-\rho_s}. \tag{10}$$

The projection matrix $A(\mathbf{0})$ is primitive, implying that every stage of the mosquito population leads to (at some point in the future) new mosquitoes in every stage. The transition matrix has the property, $\sigma(T(\mathbf{0})) < 1$, implying that in the absence of new births, the mosquito population will die out; and the property,

$$\sum_{i=1}^{\eta} T_{ij}(\mathbf{0}) \leq 1 \quad \text{for } 1 \leq j \leq \eta,$$

implying that the number of mosquitoes leaving any stage cannot be greater than the number of mosquitoes in a given stage. A straightforward application of Theorem 1.8 in Cushing (2009) [**?**], shows that the extinction equilibrium, $\mathbf{x}_e$, is globally asymptotically stable in $\bar{\mathbb{R}}_+^{\eta}$ when $R_0 < 1$.
