# Supplementary material for: Modelling the impact of insecticide-based control interventions on the evolution of insecticide resistance and disease transmission
Source: Parasit Vectors. 2018 Aug 28;11:482. doi: 10.1186/s13071-018-3025-z (PMC6114906; doi:10.1186/s13071-018-3025-z)
Supplement: Supplementary file 2 — An equivalent, intuitive, derivation of R0 for anophelene mosquitoes. (DOCX 18 kb) [file 13071_2018_3025_MOESM2_ESM.docx]

**Additional File 2.**

*Estimation of R_0_ using an ‘intuitive’ approach*

The vector population reproductive rate *R_0v_*, was defined as the number of newly emerged female adults produced by a single newly emerged female. At this point we drop the subscripts related to genotypes, and compute just for the overall population assuming it contains only a single genotype. It can be computed as follows

$$R_{0v}=\alpha E\beta\varphi\rho_{e}^{\theta_{e}}\rho_{l}^{\theta_{l}}\rho_{p}^{\theta_{p}}$$

Equation 1

The parameters in Equation 1 are as described in Table 1 of the main manuscript, with the exception of α which is the probability of a female finding a mate (assumed to be 1) and *E*, which is the expected number of broods produced by a newly emerged female. The effects of density dependence can be ignored in this calculation because *R_0v_* is defined at very low population densities when density-dependent regulation will be absent. The construction of Equation 1is therefore easily understood: the number of newly-emergent females produced by this single female is calculated as the probability that she finds a mate (α), the expected number of egg broods she lays (E), the number of female eggs in each brood $\beta\varphi$ , and the probability that each female egg survives to the newly-emerged adult stage is $\rho_{e}^{\theta_{e}}\rho_{l}^{\theta_{l}}\rho_{p}^{\theta_{p}}.$The parameter values in Equation 1 are all user-defined with the exception of the expected number of broods, *E*. It is therefore only necessary to calculate *E* to obtained R_0_ from Equation 1and *E* is calculated as follows.

The probability that a female survives and feeds on a host on day *i* of her gonadotrophic cycle, $\rho_{G}^{i}$, is found as the product of (i) the probability she has survived but remained unfed during the previous *i-1* days, i.e. $\left( \rho_{s}\left( 1-H \right) \right)^{i-1}$, and (ii) the probability that she survives and feeds on that day *i*, i.e. $\rho_{s}H$, giving

$$\rho_{G}^{i}=\left( \rho_{s}\left( 1-H \right) \right)^{i-1}\rho_{s}H$$

Equation 2

Note that this works for day *i* = 1 (i.e. day of emergence) because any number raised to the power zero is unity (e.g. $x^{i-1}=x^{0}=1$) so she is guaranteed to be alive and unfed at the start of day *i* = 1. So the overall probability of surviving to feed during her gonadotrophic cycle *p*(*f*) is the cumulative probability of completing her gonadotrophic cycle on each day

$$p\left( f \right)=\sum_{i=1}^{\infty} \left\{ \left( \rho_{s}\left( 1-H \right) \right)^{i-1}\rho_{s}H \right\}$$

Equation 3

It is possible to simplify Equation 3 by applying the algebraic result that the limit

$$\sum_{i=1}^{\infty} x^{i}=\frac{x}{1-x} if \left| x \right|<1$$

Equation 4

The ability to use this simplification makes it necessary to redefine *i* to remove the *i* -1 in Equation 3. Define the day of emergence as day 0, so that *i* = 1, 2… *n* is the number of days prior to the one under consideration. Removing day 0 from the summation gives

$$p\left( f \right)=\rho_{s}H+\sum_{i+1}^{\infty} \left\{ \left( \rho_{s}\left( 1-H \right) \right)^{i}\rho_{s}H \right\}$$

Equation 5

Or

$$p\left( f \right)=\rho_{s}H+\rho_{s}H\sum_{i+1}^{\infty} \left( \rho_{s}\left( 1-H \right) \right)^{i}$$

Equation 6

So using the algebraic technique above (Equation 4)

$$p\left( f \right)=\rho_{s}H+\rho_{s}H\frac{\rho_{s}\left( 1-H \right)}{1-\rho_{s}\left( 1-H \right)}=\rho_{s}H\left( 1+\frac{\rho_{s}\left( 1-H \right)}{1-\rho_{s}\left( 1-H \right)} \right)$$

Equation 7

After successfully finding a host and feeding with probability$p\left( f \right)$, a female will go through a non-seeking stage to complete egg maturation and oviposition (assumed to last τ-1 days where τ is the duration of her gonotrophic cycle), with daily probability of survival $\rho_{n}$, after which she has completed a gonotrophic cycle. The probability of finding a host and surviving the egg incubation period to lay eggs, $p\left( f \right)^{'}$ is therefore

$$p\left( f \right)^{'}=p\left( f \right)\rho_{n}^{\tau-1}$$

Equation 8

noting that it is necessary to use *τ*-1 because the first day of the gonotrophic cycle is the day in which she fed which is already incorporated as *p(f)*.

Female mosquitoes may go through this process more than once in their lifetime so that the expected number of broods produced by one adult female in her lifetime becomes:

$$E=p\left( f \right)^{'}+{p\left( f \right)^{'}}^{2}+{p\left( f \right)^{'}}^{3}+\ldots{p\left( f \right)^{'}}^{n}$$

Equation 9

The first term is the probability of surviving to produce one brood of eggs, the second is the probability of surviving to produce a second brood of eggs and so forth.

Equation 9 can be simplified using the above algebraic result (Equation 4) to:

$$E=\sum_{j=1}^{\infty} p{\left( f \right)'}^{j}=\frac{p\left( f \right)'}{1-p\left( f \right)'}$$

Equation 10

The values for E can then be used to solve Equation 1. This estimated vector population reproductive rate *R_0v_* allows us to predict the behaviour of the population under a given parameterisation, since the population should become extinct when *R_0v_* <1 and remain viable if $R_{0v}\geq1$.
