# Supplementary material for: Modelling the impact of insecticide-based control interventions on the evolution of insecticide resistance and disease transmission
Source: Parasit Vectors. 2018 Aug 28;11:482. doi: 10.1186/s13071-018-3025-z (PMC6114906; doi:10.1186/s13071-018-3025-z)
Supplement: Supplementary file 3 — Notes on model calibration. (DOCX 21 kb) [file 13071_2018_3025_MOESM3_ESM.docx]

**1. Model calibration for mosquito demographics.**

The model was calibrated for *Anopheles gambiae*, the most potent vector of falciparum malaria. The durations of the lifecycle stages (eggs, larvae, pupae, adult female resting between feeds) given in Table 1 of the main text were taken from refs [1, 2] [3] .

The main challenge lies in appropriate calibration for survival rates. In the laboratory, environmental conditions vary between experiments generating widely different parameter estimates [1]. In field settings, many factors such as natural heterogeneities in rainfall, wind speed, desiccation, temperature, relative humidity, density-dependence on vector abundance, and difficulties in adequate sampling procedures complicate the estimations. We therefore took the approach of using large ranges for parameter values in the sensitivity analysis (Figure 2), then demonstrating the use of the model under plausible, but somewhat arbitrary, default values of survival probabilities. These default values are provided in Table 1 and were obtained from discussions with field entomologists in Liverpool, UK; these are “consensus” values that minimized debate and should be regarded as representative but liable to change depending on local environmental factors. They are reasonably consistent with the literature. For example, daily survival probabilities of the four *Anopheles gambiae* larval instars under laboratory conditions were estimated as 0.84 ± 0.09, 0.91 ± 0.15, 0.82 ± 0.06 and 0.87 ± 0.09 respectively (our default field parameter is 0.94), and daily survival probabilities for the pupal stage as 0.76 ± 0.21 (our default field parameter is 0.55) [1]. An estimate of daily survival probability of adults conducted in the field in Kilombero, Tanzania, in a capture-recapture experiment was of 0.78 [4]. (Our default field values were 0.5 for adult male, 0.71 for host seeking females, and 0.96 for resting females).

The mating ability of the SS genotype is set to 1 and reduced mating ability of the SR and RR genotypes, due to fitness costs of carrying the resistance mutation, may be incorporated by setting $\sigma^{\mathrm{SR}}$and/or $\sigma^{RR}$ to be less than 1. We assumed all genotypes had the same mating ability.

We assume gravid females lay on average 100 eggs per oviposit. Fitness costs of resistance could be incorporated through reduced clutch size of females carrying resistance alleles, but our analyses assumed no difference between genotypes. We assume equal proportions of males and females hatch from eggs but this can potentially vary in the methodology to allow for genetic constructs which may affect sex ratio.

**2. Density-dependent mosquito population regulation.**

Mosquito population size was regulated by density dependence in the mosquito population acting at the larval stage (as in other models e.g. [2, 5]) and note that this type of population regulation is consistent with field data obtained from the Garki project, a very large African control programme [2]. More generally, it is important to identify how the mosquito population is regulated as this may affect the predicted impact of interventions [6]. Density dependence was assumed to occur at the larval stage but may have wider impact on mosquito life history traits. For example, it is known that density-dependent competition decreases survivorship and, at least in *A. gambiae,* also influences the development time, fecundity and contributes to the emergence of smaller adults, which can influence adult survival [7]. Density-dependence further impacts control strategies at lower densities because reduced competition may allow the fewer individuals to optimise traits that maximise their reproduction and survival, reducing the efficacy of the interventions [7].

The resource units have arbitrary values that define final mosquito population size. In combination, the default values gives a total female population size of ~140,000 with a roughly equal number of males (depending on their mortality rate); this is close to the ~250,000 overall population size (male and female) that we consider plausible for local deme size; the results are consistent over all population sizes, we simply selected a large one for biological realism and to reduce stochastic variation in population growth and/or resistance spread. The two other resource parameters, effect of larval competition $(c_{i}^{fj}$, $c_{i}^{mj}$) and relative resource consumption $(\omega_{i}^{fj}$, $\omega_{i}^{mj}$), can vary between genotypes to reflect differences such as fitness costs of resistance in terms of competitive ability; we ignored these potential differences in our simulations and the parameter values were identical for all genotypes. We would suggest the most competitive and/or resource-hungry genotype be set to 1 and the other genotypic values all relative to this so that the values lie in the range 0 to 1; we used entirely arbitrary values of 0.67 for each.

**3. Estimating mosquito density and other parameters in the Ross-Macdonald model.**

The Ross-Macdonald model used in the main text requires an estimate of mosquito density i.e. number of female mosquitoes per human host, *m*. Two values for *m* were calculated to reflect intense transmission of *Plasmodium falciparum* by *Anopheles gambiae*. The first estimate was obtained from the capture-recapture trial described in [4]. They found the highest number of mosquitoes inside a house was 900 per day so, assuming that the average number of humans per house is eight, gives *m* = 900/8 =113. The second estimate came from Churcher *et al.* [8] who reported an annual entomological inoculation rate of ~200 and *A. gambiae* sporozoite rate of ~2% in a study site in Senegal. This implies 10,000 bites per person per year which, assuming mosquitoes bite every four days, is equivalent to m=(10,000/365)*4≈110. Hereafter we will use a value of m=110 as an approximation for high transmission settings. The default settings in Table 1 of the main text result in an equilibrium female mosquito population size of 135,878 which corresponds to a human population size of 135,878/110= 1,235 which can be used in Equation 19 of the main text to gauge the impact of control measures reducing adult female population size (e.g. Table 2 of main text).

The other parameters in the Ross-Macdonald model (i.e. *a,* $b_{1}$*,* $b_{2}$, and *r*) are all consensus values for *A. gambiae* taken from Refs. [9, 10]. Note that the rate at which humans recover from a malaria infection is 0.01 which equates to a mean duration of human infection of 100 days.

References

1. Olayemi IK, Ande AT. Life table analysis of *Anopheles gambiae* (diptera: culicidae) in relation to malaria transmission. J Vector Borne Dis. 2009;46:295-8.

2. White M, Griffin J, Churcher T, Ferguson N, Basanez M-G, Ghani A. Modelling the impact of vector control interventions on *Anopheles gambiae* population dynamics. Parasit Vectors. 2011;4:153.

3. Chitnis N, Schapira A, Smith T, Steketee R. Comparing the effectiveness of malaria vector-control interventions through a mathematical model. Am J Trop Med Hyg. 2010;83:230-40.

4. Takken W, Charlwood JD, Billingsley PF, Gort G. Dispersal and survival of  *Anopheles funestus* and *A. gambiae* s.l. (Diptera: Culicidae) during the rainy season in southeast Tanzania. Bull Entomol Res. 1998;88:561-6.

5. Hancock P, Godfray HC. Application of the lumped age-class technique to studying the dynamics of malaria-mosquito-human interactions. Malar J. 2007;6:98.

6. Russell TL, Lwetoijera DW, Knols BGJ, Takken W, Killeen GF, Ferguson HM. Linking individual phenotype to density-dependent population growth: the influence of body size on the population dynamics of malaria vectors. Proc R Soc Lond B. 2011;278:3142-51;

7. Gimnig JE, Ombok M, Otieno S, Kaufman MG, Vulule JM, Walker ED. Density-dependent development of *Anopheles gambiae* (Diptera: Culicidae) larvae in artificial habitats. J Med Entomol. 2002;39:162-72.

8. Churcher TS, Trape JF, Cohuet A. Human-to-mosquito transmission efficiency increases as malaria is controlled. Nat Commun. 2015;6:6054.

9. Smith D, McKenzie EF. Statics and dynamics of malaria infection in Anopheles mosquitoes. Malar J. 2004;3:13.

10. Menach A, McKenzie FE, Flahault A, Smith D. The unexpected importance of mosquito oviposition behaviour for malaria: non-productive larval habitats can be sources for malaria transmission. Malar J. 2005;4:23.
